# Regulation of B-cell development and tolerance by different members of the miR-17∼92 family microRNAs

**DOI:** 10.1038/ncomms12207

**Published:** 2016-08-02

**Authors:** Maoyi Lai, Alicia Gonzalez-Martin, Anthony B. Cooper, Hiroyo Oda, Hyun Yong Jin, Jovan Shepherd, Linling He, Jiang Zhu, David Nemazee, Changchun Xiao

**Affiliations:** 1 Department of Immunology and Microbial Science, The Scripps Research Institute, 10550 North Torrey Pines Road, La Jolla, California 92037, USA; 2 Kellogg School of Science and Technology, The Scripps Research Institute, La Jolla, California 92037, USA

## Abstract

The molecular mechanisms that regulate B-cell development and tolerance remain incompletely understood. In this study, we identify a critical role for the miR-17∼92 microRNA cluster in regulating B-cell central tolerance and demonstrate that these miRNAs control early B-cell development in a cell-intrinsic manner. While the cluster member miR-19 suppresses the expression of *Pten* and plays a key role in regulating B-cell tolerance, miR-17 controls early B-cell development through other molecular pathways. These findings demonstrate differential control of two closely linked B-cell developmental stages by different members of a single microRNA cluster through distinct molecular pathways.

## Abstract (web summary)

MicroRNA cluster 17∼92 plays a critical role in B-cell differentiation. Here the authors show that miR-19 regulates B-cell tolerance via suppressing the expression of *PTEN*, whereas miR- 17 is essential for early B-cell development independently of Pten, Phlpp2, or Bim.

## Graphical abstract

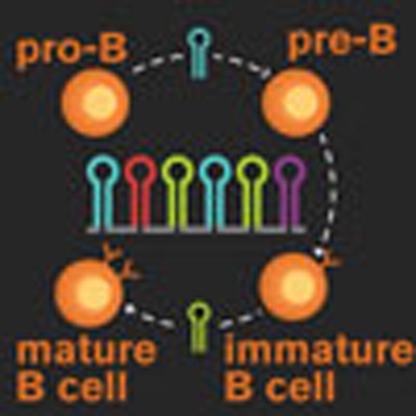

A defining feature of B-cell development is the process of gene rearrangements in the B-cell receptor (BCR) loci, through which B cells acquire the capacity to express a BCR of a given specificity on the cell surface^1^. These rearrangements occur in an orderly manner over time, sequentially involving the immunoglobulin heavy (IgH) and light (IgL) chain genes during the pro-B and the pre-B stages of B-cell development, respectively. This is thought to depend on an orderly accessibility of the corresponding loci to the RAG1 and 2 recombinases, which mediate rearrangements of variable (*V*), diverse (*D*), and joining (*J*) gene segments through the process called *V*(*D*)*J* recombination. At the end of this process, each immature B cell expresses a single IgH and a single IgL chain, with a single and, most times, unique antigen specificity. The combinatorial and stochastic nature of gene rearrangements leads to the generation of immature B cells with self-reactive receptors. At this immature stage, the newborn B cells undergo the first checkpoint for self-reactivity, to eliminate potentially autoreactive cells by central tolerance mechanisms. Once a newborn B cell encounters a self-antigen for which its BCR is specific, it attempts to escape autoreactivity by continuing *V*(*D*)*J* recombination at the IgL locus (receptor editing) or dying by apoptosis (clonal deletion). When the cell has passed this developmental checkpoint, it differentiates into a mature B cell. Self-reactive B cells can be further regulated in the periphery through peripheral tolerance mechanisms, including the induction of anergy^2^,^3^. Previous studies of human B cells showed that self-reactivity is progressively diminished during normal B-cell development, consistent with the idea that several tolerance mechanisms are at work at different stages of the life of B cells^4^.

Despite intensive study, our understanding of molecular pathways regulating B-cell development and tolerance is still incomplete. Specifically, the function of individual microRNAs (miRNAs) in B-cell development and tolerance remains poorly understood. miRNAs are endogenously encoded single-stranded RNAs of ∼22 nucleotides in length. To date, ∼2,500 human and ∼1,900 mouse miRNAs have been identified and many of them play essential roles in the immune system^5^,^6^,^7^. They regulate gene expression by pairing with messenger RNAs through imperfect sequence complementarity, resulting in reduced protein output by mRNA cleavage, translational repression or promotion of mRNA decay^8^. It has been estimated that 25∼40% miRNA precursors are located in close proximity (<10 kb) of other miRNA precursors, constituting miRNA clusters^9^,^10^,^11^. The majority of miRNA clusters are first transcribed into single polycistronic primary transcripts (pri-miRNAs) and then cleaved by Drosha into individual hairpins (pre-miRNAs), which are further processed by Dicer to produce mature miRNAs. Gene expression profiling studies have shown that the expression of different miRNAs in a cluster is generally co-regulated^12^,^13^, suggesting that they may cooperate with each other to accomplish common functions. Furthermore, comparative genomics show that miRNA clusters are evolutionarily stable and conserved across species, suggesting functional importance of such organization^14^,^15^. Some clusters consist of miRNAs with identical seed regions (termed homogeneous miRNA clusters), probably a result of gene duplication. The regulatory effect of homogeneous miRNA clusters may simply be an increase in gene dosage. Other clusters are composed of miRNAs with different seed regions (termed heterogeneous miRNA clusters). It remains unclear how members of heterogeneous miRNA clusters operate together to accomplish common functions^15^.

In this study we dissected the roles of the miRNA-17∼92 family miRNAs at different stages of B-cell development. The miR-17∼92 family consists of three miRNA clusters: miR-17∼92, miR-106a∼363 and miR-106b∼25. Together, these three clusters contain 15 miRNA stem loops that give rise to 13 distinct mature miRNAs, which fall into four miRNA subfamilies (miR-17, miR-18, miR-19 and miR-92 subfamilies), with members in each subfamily sharing the same seed sequence^16^. The genomic organization and mature miRNA sequences of this family are conserved in all vertebrates^17^. During lymphocyte development, these miRNAs are highly expressed in progenitor cells, with expression levels decreasing two- to threefold on maturation^13^,^18^. Mouse genetic studies showed that miR-17∼92-deficient mice were runted and died at birth due to lung and heart hypoplasia. In the haematopoietic system, there was a partial block of early B-cell development at the pro- to pre-B transition. Deletion of miR-106a∼363 and miR-106b∼25 has no obvious phenotypic consequences, whereas compound mutant embryos lacking both miR-17∼92 and miR-106b∼25 died at midgestation, suggesting functional redundancy between these miRNA clusters^19^. Conversely, overexpression of miR-17∼92 family miRNAs occurs frequently in a broad spectrum of human cancers and in lymphocytes from patients with autoimmune diseases^16^,^20^,^21^,^22^,^23^. These observations suggest that miR-17∼92 is involved in lymphoma development and autoimmune diseases. We have generated a conditional miR-17∼92 transgenic allele (termed miR-17∼92 Tg) whose expression can be turned on by Cre recombinase^24^. When miR-17∼92 Tg was turned on specifically in B cells using CD19-Cre, transgenic mice exhibited premature death^25^. About 80% of those mice developed lymphomas, demonstrating that miR-17∼92 is a powerful cancer driver^25^. Notably, the other 20% miR-17∼92 transgenic mice died of autoimmune diseases. We speculate that miR-17∼92 might play a critical role in the control of B-cell tolerance. In this study, we use two newly generated *in vivo* models to investigate the function and mechanism of miR-17∼92 family miRNAs in regulating B-cell development and tolerance, and demonstrate functional specificity of different members of this cluster at two closely linked developmental stages of B cells.

## Results

### miR-17∼92 regulates B-cell central tolerance

**Figure 1:**
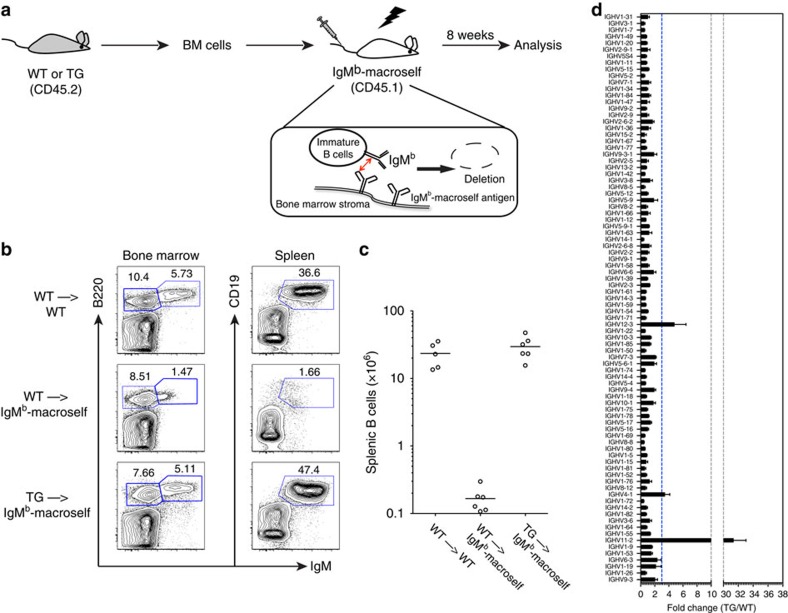
miR-17∼92 controls B-cell central tolerance. (**a**) Outline of bone marrow reconstitution experiment. In the bone marrow of IgM^b^-macroself recipient mice, all nascent immature B cells undergo negative selection as a result of the interaction between the surface IgM and the ubiquitously expressed anti-IgM superantigen. (**b**) Representative flow cytometry plots showing B-cell development in the bone marrow and spleen of recipient mice. (**c**) Numbers of donor-derived splenic B cells (CD45.2^+^CD19^+^IgM^+^) in bone marrow-reconstituted mice, with each dot representing a single mouse and the horizontal bar indicating the average cell number for each group. (**d**) BCR repertoire analysis based on *IGHV* gene usage in splenic B cells from WT C57BL/6J (WT) and CD19Cre;miR-17∼92 transgenic (TG) mice. B cells were activated *in vitro* to facilitate the analysis. Results are presented as fold change of TG over WT mice. The dotted line was set at the arbitrary value of 3, to indicate *IGHV* genes with more than threefold increase in usage in TG mice as compared with WT mice. Data are representative of three (**b**,**c**) and two independent experiments (**d**) (mean±s.e.m. in **d**) with *n*=5 (WT to WT) or 6 (WT to IgM^b^-macroself and TG to IgM^b^-macroself) in **b**,**c** and *n*=3 in **d**.

To analyse the effect of transgenic miR-17∼92 expression on B-cell central tolerance, we used the recently established IgM^b^-macroself mouse model^26^. In this model, mice are engineered to ubiquitously express a superantigen reactive to the heavy chain constant region of IgM, the first BCR expressed on the surface of immature B cells. As receptor editing changes only the light chain, it fails to eliminate superantigen reactivity and all developing B cells undergo cell death by clonal deletion. As a consequence, these mice have almost no mature B cells in the spleen and lymph nodes. We analysed B-cell development in IgM^b^-macroself recipients (with surface marker CD45.1^+^) reconstituted with bone marrow from CD19-Cre;miR-17∼92 Tg/Tg (TG) or wild-type (WT) mice (with surface marker CD45.2^+^) (Fig. 1a). The WT→IgM^b^-macroself chimeras exhibited a severe B-cell lymphopenic phenotype recapitulating that of the IgM^b^-macroself mice, characterized by a complete developmental block at the immature B-cell stage in the bone marrow that results in the absence of mature B cells in the spleen. Strikingly, B-cell development in the TG→IgM^b^-macroself chimeras was similar to that in WT→WT chimeras (Fig. 1b,c). Consistent with the restoration of peripheral B cells, serum IgM levels were also substantially restored in the TG→IgM^b^-macroself chimeras (Supplementary Fig. 1a). Moreover, these rescued B cells appeared to be functional, as TG→IgM^b^-macroself chimeras mounted a close-to-normal antibody response upon NP-CGG immunization (Supplementary Fig. 1b). This is the first time that we observed such a complete restoration of B-cell development in the IgM^b^-macroself central tolerance model by a miRNA gene.

Central tolerance, when functionally intact, protects against autoimmunity by purging a large majority of self-reactive B cells from the B-cell compartment^2^,^3^. As B cells with elevated miR-17∼92 expression were able to escape from central tolerance in IgM^b^-macroself model, in which all B cells are self-reactive, we reasoned that the BCR repertoire in TG mice might be significantly different from that of WT mice. To assess this, we analysed the BCR repertoire by performing deep sequencing of Ig heavy chain *V* genes (*IGHV*) of WT and TG mice. As predicted, TG mice exhibited an IGHV landscape different from that of WT mice, with drastically increased usage in TG mice (>3-fold) of three *IGHV* genes (*IGHV12-3*, *IGHV4-1* and *IGHV11-2*) (Fig. 1d). Among these, V_H_11 and V_H_12 gene families are predominantly associated with germline-encoded autoantibodies of phosphatidylcholine specificities^27^,^28^. A large numbers of V_H_11^+^ B cells, which are rarely detected in the spleen of WT mice, were present in the spleen of TG mice, as detected by using an anti-idiotypic monoclonal antibody for V_H_11 (Supplementary Fig. 1c). Conversely, we found reduced usage of IGHV11-2 and IGHV12-3 in B cells deficient for the miR-17∼92 miRNA family (CD19tKO) (Supplementary Fig. 1d). In addition, we measured the presence of anti-double-stranded DNA autoantibodies in the serum of WT and TG mice at terminal analysis. High titres of autoantibodies were detected in 4 out of 45 TG mice, but were not found in any of the WT mice (Supplementary Fig. 1e).

We next investigated the function of physiological levels of miR-17∼92 family miRNAs in B-cell central tolerance. We analysed receptor editing in WT mice and mice with B-cell-specific deletion of this miRNA family (by either Mb1-Cre or CD19-Cre, termed Mb1tKO and CD19tKO mice, respectively). Deletion of the miR-17∼92 family miRNAs in B cells impaired receptor editing, as indicated by increased κ/λ-light chain ratios and reduced percentages of λ-light chain-positive (Igλ^+^) immature B cells (Supplementary Fig. 2a–d). Conversely, transgenic miR-17∼92 expression promoted receptor editing (Supplementary Fig. 2c–d). These results show that endogenous levels of miR-17∼92 family miRNAs exquisitely regulate receptor editing at the B-cell central tolerance checkpoint. We further analysed the effect of transgenic miR-17∼92 expression on B-cell central tolerance in the IgHEL;mHEL mouse model^29^. In this model, IgHEL mice express a transgenic BCR that is specific for hen egg lysozyme (HEL) in their B cells, whereas mHEL mice express a membrane-bound HEL in a wide range of cells. In IgHEL;mHEL double transgenic mice, developing B cells are deleted in the bone marrow and few mature B cells appear in the peripheral lymphoid organs^30^. Consistent with the previous report, we detected very small numbers of B cells bearing IgHEL (IgD^a+^) in the spleens of mHEL mice reconstituted with bone marrow cells from WT IgHEL mice (WT; IgHEL). The number of splenic IgHEL (IgD^a+^) B cells increased by fivefold when mHEL mice were reconstituted with bone marrow cells from IgHEL mice with B-cell-specific transgenic miR-17∼92 expression (TG;IgHEL) (Supplementary Fig. 3). These data, together with previous results from the IgM^b^-macroself model (Fig. 1b,c), demonstrate a general effect of elevated miR-17∼92 expression on B-cell selection against self-antigens. Therefore, our data show that miR-17∼92 is a critical regulator of B-cell central tolerance at the immature B-cell stage.

### miR-19 plays a key role in B-cell central tolerance

**Figure 2:**
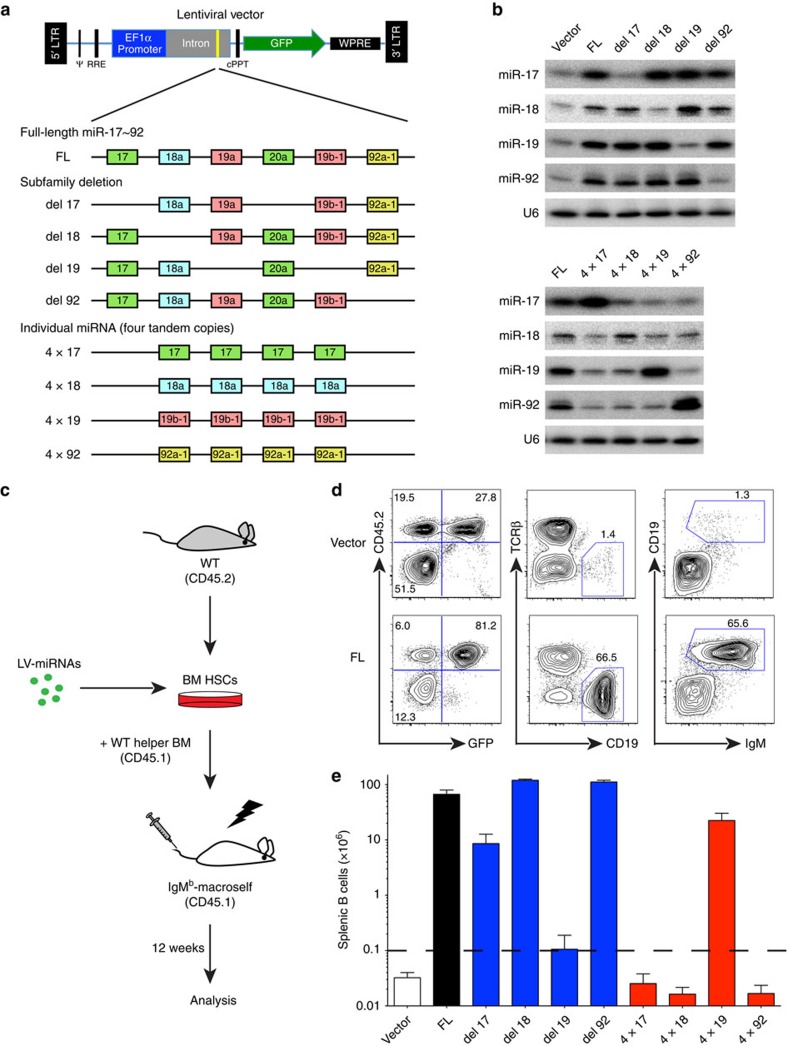
miR-19 plays a critical role in regulating B-cell central tolerance. (**a**) Lentiviral vectors expressing the FL miR-17∼92, its deletion mutants and individual members. miRNAs sharing the same seed region belong to the same subfamily and are depicted in the same colour. (**b**) Northern blot analysis of miRNA expression in HeLa cells transduced with indicated lentiviruses. (**c**) Outline of experimental strategy. HSCs were enriched from WT donors to facilitate the transduction by lentiviruses encoding various combinations of miR-17∼92 miRNAs (LV-miRNAs). (**d**) Representative flow cytometry plots of lymphocytes from the spleen of recipient mice. It is noteworthy that lentiviruses encoding miR-17∼92 (FL) fully rescued the B-cell compartment, and that virus transduced cells (CD45.2^+^GFP^+^) outcompeted WT helper-derived cells (CD45.2^−^GFP^−^) and non-transduced cells (CD45.2^+^GFP^−^). (**e**) Numbers of splenic B cells in IgM^b^-macroself recipient mice. B-cell number above the dash line indicates break of tolerance. Data are representative of 3 (**b**) or 8 (**d**) or pooled from 8 independent experiments (**e**) (mean±s.e.m. in **e**) with *n*=5 (Vector) or 14 (FL) in **d**, and *n*=5 (Vector and del18), 14 (FL), 10 (del17 and 4 × 19), 4 (del19 and del92) or 6 (4 × 17, 4 × 18 and 4 × 92) in **e**.

We next investigated the functional contribution of individual miR-17∼92 members in regulating B-cell central tolerance. The six miRNAs of miR-17∼92 fall into four miRNA subfamilies (the miR-17, miR-18, miR-19 and miR-92 subfamilies), with members in each subfamily sharing the same seed region and probably similar functions. We generated two groups of lentiviral vectors expressing either the miR-17∼92 cluster with one miRNA subfamily deleted, or four tandem copies of individual miRNAs (Fig. 2a). Northern blot analyses confirmed that these lentiviral vectors expressed encoded miRNAs at levels comparable to a lentiviral vector encoding the full-length (FL) miR-17∼92 cluster (Fig. 2b). Highly enriched CD45.2^+^ WT haematopoietic stem cells (HSCs) were transduced with lentiviruses encoding various combinations of miR-17∼92 miRNAs and were used to reconstitute lethally irradiated IgM^b^-macroself recipients (CD45.1^+^), together with unmanipulated WT bone marrow cells (CD45.1^+^) (Fig. 2c). Twelve weeks after reconstitution, the bone marrow chimeras were analysed for splenic B cells. Although chimeras reconstituted with control lentivirus-transduced HSCs exhibited the same B-cell lymphopenic phenotype as unmanipulated IgM^b^-macroself mice, lentiviral expression of miR-17∼92 restored the B-cell compartment in IgM^b^-macroself recipient mice, similar to that by TG bone marrow cells (Figs 1b,c and 2d,e). The contribution of each miR-17∼92 miRNAs to the regulation of B-cell tolerance was determined by quantifying the numbers of splenic B cells that escaped IgM^b^-macroself-mediated deletion. As shown in Fig. 2e, deletion of the miR-19 subfamily almost completely abrogated the escape of B cells, whereas miR-17 subfamily deletion caused a modest reduction in splenic B-cell numbers. Deletion of miR-18 or miR-92 did not alter the ability of miR-17∼92 to restore B-cell development in IgM^b^-macroself recipient mice. Consistently, lentiviral expression of miR-19 alone restored B-cell development substantially, whereas other individual miR-17∼92 miRNAs did not have any significant effect (Fig. 2e). Based on these observations, we conclude that miR-19 subfamily miRNAs are the key members of the miR-17∼92 cluster in regulating B-cell central tolerance, whereas miR-17 subfamily miRNAs play a supporting role in this process.

### miR-19 controls B-cell central tolerance by suppressing *Pten*

Our previous photoactivatable-ribonucleoside-enhanced cross-linking and immunoprecipitation analyses of mature B cells identified 868 protein-coding genes that contain miR-17∼92 miRNA-binding sites conserved in humans and mice^25^. To gain new insights into the molecular mechanisms by which miR-17∼92 regulates B-cell central tolerance, we focused on target genes containing binding sites for miR-19, the critical member of miR-17∼92 in this process. The miR-19 targetome contains 386 protein-coding genes with 404 binding sites. These genes function in a broad spectrum of biological processes. Among them, regulators of the phosphatidylinositol-3 kinase (PI3K) pathway (phosphatase and tensin homologue (PTEN) and Phlpp2) and apoptosis pathway (Bim) are of special interest. The PI3K pathway plays a critical role in supporting B-cell survival at the mature B-cell stage^31^, whereas the apoptosis pathway is thought to determine the cell fate during B-cell central tolerance^32^,^33^.

**Figure 3:**
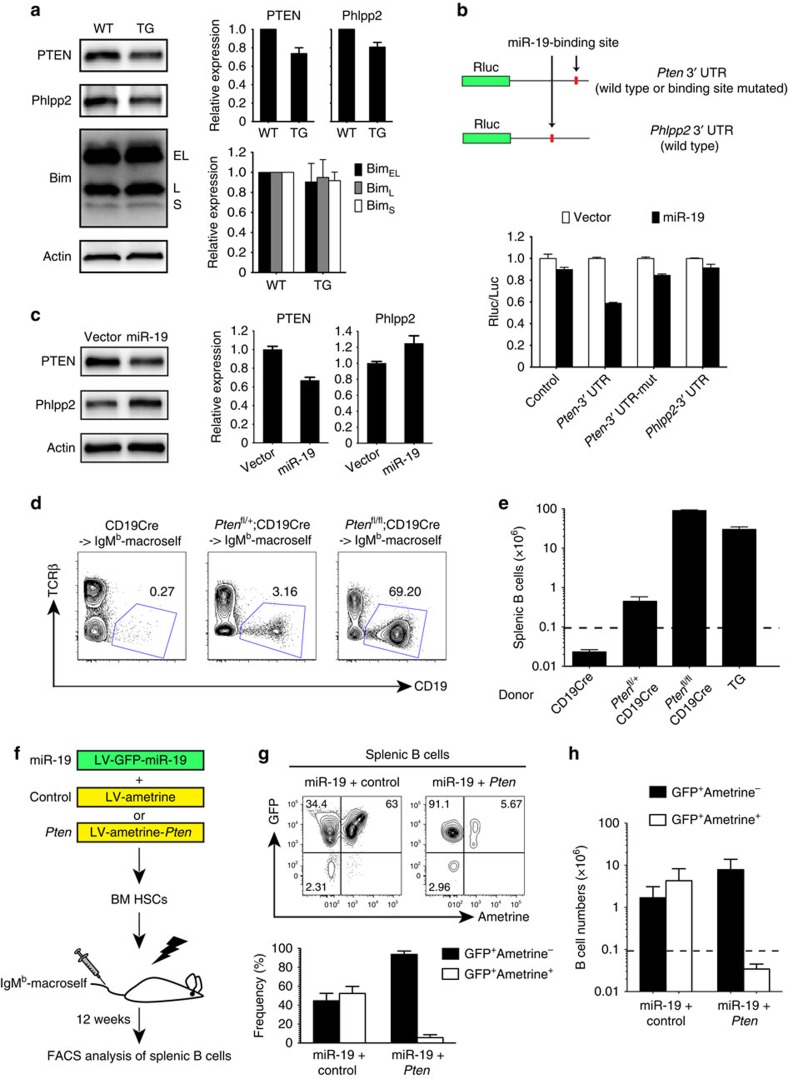
PTEN mediates miR-19 control of B-cell central tolerance at the immature B-cell stage. (**a**) Western blot analysis of PTEN, Phlpp2 and Bim protein levels in *in vitro*-cultured immature B cells from ∼7-week-old WT and TG mice. Expression levels were normalized to Actin. (**b**) Reporter assays of *Rluc* gene expression containing the *Pten* or *Phlpp2* 3′-UTR fragments harbouring predicted miR-19-binding sites at indicated locations. HeLa cells were co-transfected with luciferase reporter and miR-19-expressing or control vector. The Rluc/Luc activity ratio was arbitrarily set as 1 for control vector for each reporter. The miR-19-binding site was mutated in *Pten*-3′-UTR-mut. (**c**) Western blot analysis of PTEN and Phlpp2 protein levels in HeLa cells transduced with miR-19-expressing (miR-19) or control lentiviruses (vector). Protein levels were normalized to Actin. (**d**) Representative flow cytometry plots showing spleen B-cell compartment of recipient mice. Donor genotypes are indicated above each plot. (**e**) Numbers of splenic B cells in IgM^b^-macroself mice reconstituted with bone marrow cells from mice of indicated genotypes. (**f**) Outline of experimental strategy. HSCs enriched from WT donors were transduced with lentiviruses expressing miR-19 and GFP (miR-19) together with lentiviruses expressing *Pten* and Ametrine (*Pten*) or Ametrine alone (Control), and used to reconstitute IgM^b^-macroself mice, which were analysed for splenic B cells 12 weeks later. (**g**) Representative flow cytometry plots of escaped B cells and bar graphs summarizing the frequencies of GFP^+^Ametrine^−^ and GFP^+^Ametrine^+^ cells among lentivirus-transduced B cells. (**h**) Numbers of splenic B cells in IgM^b^-macroself recipient mice. GFP^+^Ametrine^−^ cells were from HSCs transduced with miR-19-expressing lentiviruses alone, whereas GFP^+^Ametrine^+^ cells were from HSCs transduced with miR-19-expressing lentiviruses together with lentiviruses expressing *Pten* and Ametrine (*Pten*) or Ametrine alone (Control). Data are representative of 3 (**a**,**g**) or 2 (**c**) or 3 (**d**) or pooled from 4 (**b**,**e**), 2 (**c**) or 3 (**g**,**h**) independent experiments (mean±s.e.m. in **a**,**b**,**c**,**e**,**g**,**h**) with *n*=3 (CD19Cre), 5 (Pten^fl/+^CD19Cre) or 6 (Pten^fl/fl^CD19Cre) in **d**,**e** and *n*=7 (miR19+Control) or 8 (miR-19+Pten) in **g**,**h**. B-cell numbers above the dash line indicates break of tolerance (**e**,**h**).

To assess whether miR-17∼92 regulates the expression of these target genes in immature B cells, we purified B220^+^IgM^+^CD93^+^IgD^−^ immature B cells from an *in vitro* culture of bone marrow B-cell precursors (Supplementary Fig. 4) and measured protein expression by immunoblot analysis. Expression of PTEN and Phlpp2 in TG immature B cells was reduced to 70% and 80% of WT levels, respectively. The expression levels of Bim were reduced marginally and this reduction did not reach statistical significance (Fig. 3a). To determine whether miR-19 directly binds to the *Pten* and *Phlpp2* mRNAs, we performed reporter assays with a *Renilla* luciferase (Rluc) reporter gene containing the *Pten* and *Phlpp2* 3′-untranslated region (UTR) fragments encompassing the predicted miR-19 binding sites. Overexpression of miR-19 decreased the Pten reporter activity by >40%, but did not reduce the Phlpp2 reporter activity (Fig. 3b). Mutation of the miR-19 binding site in the *Pten* 3′-UTR abrogated the inhibitory effect of miR-19, further confirming that miR-19 directly regulates *Pten* expression. Consistently, miR-19 overexpression significantly reduced endogenous PTEN, but not Phlpp2, protein levels in HeLa cells (Fig. 3c).

To evaluate whether the reduction in PTEN protein levels contributes to miR-17∼92 regulation of B-cell tolerance, we reconstituted lethally irradiated IgM^b^-macroself mice with bone marrow from *Pten*-deficient mice. Heterozygous deletion of *Pten* (*Pten*^fl/+^;CD19-Cre) resulted in significant restoration of the B-cell compartment in IgM^b^-macroself recipients (Fig. 3d,e). Remarkably, homozygous deletion of the *Pten* gene (*Pten*^fl/fl^; CD19-Cre) completely restored the B-cell compartment, which is comparable to TG→IgM^b^-macroself chimeras (Fig. 3d,e). We next used the lentiviral expression system to co-express miR-19 and *Pten* in HSCs, as illustrated in Fig. 3f, to test whether restoring *Pten* expression can prevent the break of B-cell central tolerance by miR-19 in the IgM^b^-macroself recipients. The miR-19-expressing lentivirus encodes GFP, whereas the *Pten*-expressing lentivirus encodes Ametrine, therefore allowing distinguishing cells transduced by a single lentivirus from those transduced by both. Terminal analysis of the reconstituted mice showed no difference in the total numbers of splenic B cells that escaped the central tolerance checkpoint, whether *Pten* or its vector control was introduced to the miR-19 overexpression scheme. However, we observed a drastic difference in the compositions of these escaped B cells. In the miR-19+control group, the number of B cells expressing miR-19 alone (GFP^+^Ametrine^−^) was comparable to that of B cells co-expressing miR-19 and control (GFP^+^Ametrine^+^). In the miR-19+*Pten* group, the escaped B cells were predominantly those expressing miR-19 alone (GFP^+^Ametrine^−^), whereas the number of B cells co-expressing miR-19 and *Pten* (GFP^+^Ametrine^+^) was close to the IgM^b^-macroself background level (Fig. 3g,h). Taken together, these results suggest a miR-19-*Pten* pathway that regulates B-cell central tolerance.

### Impaired B-cell development in the absence of miR-17∼92

**Figure 4:**
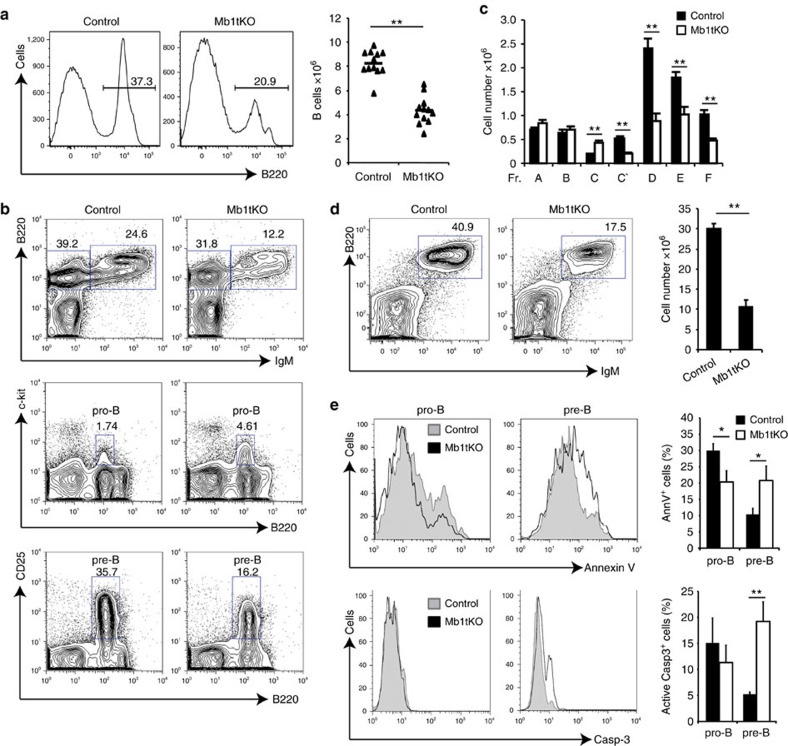
Complete deletion of the miR-17∼92 miRNA family impairs early B-cell development. (**a**) B-cell percentages in the bone marrow of representative control and Mb1tKO mice are shown in the histograms and B-cell numbers in the bone marrow of all mice analysed are shown in the graph. (**b**) Representative plots of IgM^+^ B cells (B220^+^IgM^+^), pro-B cells (B220^int^ckit^+^) and pre-B cells (B220^int^CD25^+^) in the bone marrow of control and Mb1tKO mice. (**c**) Cell numbers of all B-cell developmental stages in the bone marrow (Hardy fractions A to F) in control and Mb1tKO mice. (**d**) B-cell percentages in the spleen of representative control and Mb1tKO mice are shown in the histograms and splenic B-cell numbers are shown in the graph. (**e**) Apoptosis of pro-B and pre-B cells was measured by flow cytometry analysis of annexin V and active caspase 3. *P<0.05 and **P<0.01 (two-tailed Student's *t*-test). Data are representative of 2 (**a**,**b**,**d**) or pooled from 2 (**c**,**d**,**e**: active caspase 3) or 3 (**e**: Annexin V) independent experiments (mean±s.e.m. in **c**,**d**,**e**) with *n*=12 (Control and Mb1tKO) in **a**–**d**, 8 (Control and Mb1tKO) in **e** (Annexin V) and 5 (Control and Mb1tKO) in **e** (active caspase 3).

A previous study showed that germline deletion of miR-17∼92 led to a partial block of early B-cell development at the pro- to pre-B transition and Bim was suggested to be a key functional target of miR-17∼92 in this process^19^. However, another study reported that transgenic mice with ubiquitous overexpression of miR-17, a member of the miR-17∼92 cluster, exhibited overall growth retardation and severely reduced numbers of B lineage cells in both the bone marrow and the spleen^34^. Therefore, the cell autonomous function of the miR-17∼92 family, as well as the functional contribution of individual miRNAs of this family, in B-cell development remains to be elucidated. To investigate the cell-intrinsic role of miR-17∼92 family miRNAs in B-cell development, we generated Mb1-Cre;miR-17∼92^fl/fl^; miR-106a∼363^−/−^;miR-106b∼25^−/−^ mice (termed Mb1tKO). In these mice, miR-17∼92 is deleted specifically in the B-cell lineage by Mb1-Cre, a Cre allele expressed from the earliest stage of B-cell development^35^, whereas the two paralogue clusters, miR-106a∼363 and miR-106b∼25, are deleted in the germline, resulting in a complete absence of this family in the B-cell lineage. Analysis of Mb1tKO mice revealed a twofold reduction in B-cell numbers in the bone marrow (Fig. 4a). Consistent with the previous study of miR-17∼92 germline knockout mice, Mb1tKO mice showed an accumulation of pro-B cells (B220^int^cKit^+^) and a reduction of pre-B cells (B220^int^CD25^+^) (Fig. 4b,c). The splenic B-cell number in Mb1tKO mice was also reduced by threefold (Fig. 4d). Apoptosis of pro-B and pre-B cells of Mb1tKO mice was determined by flow cytometry analysis of Annexin V and active caspase 3. We observed an increased apoptotic rate in pre-B cells of Mb1tKO mice when compared with their counterparts in WT mice (Fig. 4e). In conclusion, the miR-17∼92 family miRNAs control the pro- to pre-B transition during B-cell development in a cell-autonomous manner and the complete absence of this miRNA family leads to increased apoptosis of pre-B cells.

### A central role of miR-17 in early B-cell development

**Figure 5:**
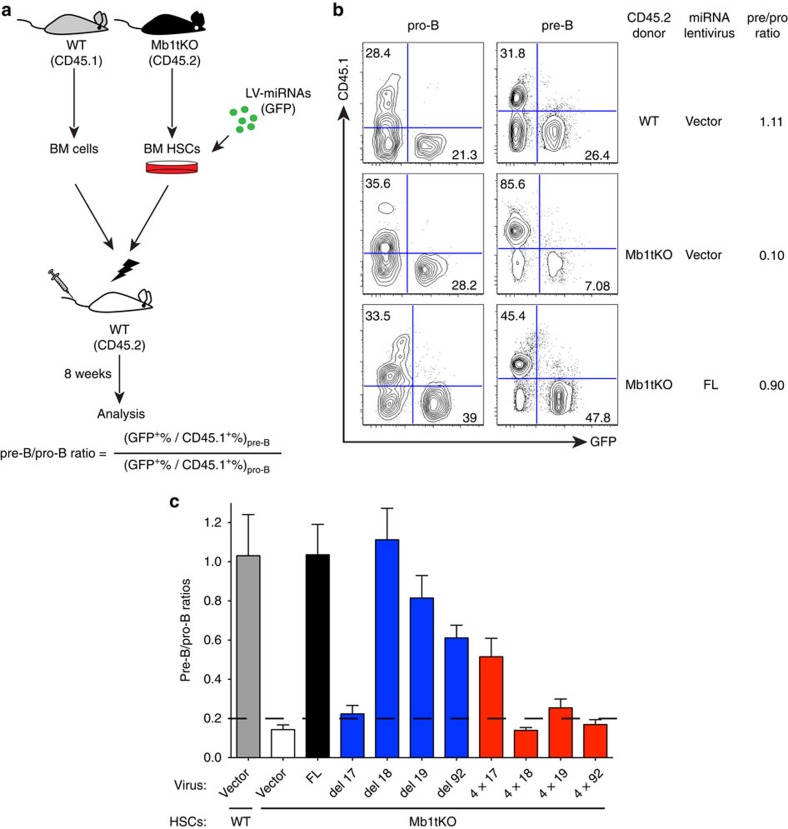
miR-17 plays a central role in regulating the pro-B to pre-B transition. (**a**) Outline of experimental strategy. HSCs were enriched from the Mb1tKO donors, transduced with miRNA-expressing lentiviruses and mixed with bone marrow cells from the congenic WT mice, to generate mixed bone marrow chimeras. Reconstituted mice were analysed 8 weeks later. Ratios of lentivirus-transduced cells (GFP^+^) versus the WT donor-derived cells (CD45.1^+^) in pro-B cells (B220^+^cKit^+^) and pre-B cells (B220^+^CD25^+^) were quantified. (**b**) Representative flow cytometry plots of pro-B cells and pre-B cells in the bone marrow of recipient mice of indicated groups, showing contributions from WT-derived cells (CD45.1^+^) and lentivirus-transduced cells (GFP^+^). A reconstitution group with control virus-transduced CD45.2^+^ WT HSCs mixed with the CD45.1^+^ WT BM cells is included for comparison. (**c**) Changes in ratios of GFP^+^ cells versus CD45.1^+^ cells during the pro-B to pre-B transition. The dash line marks the pre-B/pro-B ratio in Mb1tKO mice. Data are representative (**b**) or pooled (**c**) from 6 independent experiments (mean±s.e.m. in **c**) with *n*=3 (WT-Vector), 8 (tKO-Vector and 4 × 17), 11 (FL), 7 (del17 and 4 × 19), 4 (del18, del19 and del92) or 6 (4 × 18 and 4 × 92) in **b**,**c**.

We next analysed which individual miRNA subfamily of the miR-17∼92 cluster plays an essential role in the pro- to pre-B-cell transition. To evaluate the functional contribution of each miRNA subfamily of the miR-17∼92 cluster to the regulation of early B cell development, we restored the expression of miR-17∼92 miRNAs, either individually or in combination (Fig. 2a), in Mb1tKO HSCs by lentiviral transduction and determined their ability to undergo the pro- to pre-B-cell transition. A mixed bone marrow reconstitution approach, in which Mb1tKO B cells were competing with their WT counterparts during B-cell development, was performed to highlight the B-cell developmental block of Mb1tKO mice (Fig. 5a). In this experimental setting, the changes in the ratio of virus-transduced CD45.2^+^ cells (GFP^+^) versus WT competitors (CD45.1^+^) during the pro- to pre-B transition provided a measurement for the developmental defect. In the control WT:WT mix group, as no major difference existed between these two populations, the GFP^+^/CD45.1^+^ ratios remained unchanged during the pro- to pre-B transition, resulting in a pre-B/pro-B ratio close to 1 (Fig. 5b upper panels and Fig. 5c). In contrast, cells derived from the Mb1tKO HSCs, when transduced with control virus, underwent a severe developmental block in competition with WT cells. Therefore, the GFP^+^/CD45.1^+^ ratio shifted drastically as WT cells became dominant in the pre-B population, resulting in a pre-B/pro-B ratio below 0.2 (Fig. 5b middle panels and Fig. 5c). As expected, when Mb1tKO cells were transduced with virus expressing the FL miR-17∼92, they became fully competitive with WT cells and the GFP^+^/CD45.1^+^ ratio in pre-B cells was comparable to that in pro-B cells, resulting in a pre-B/pro-B ratio close to 1 (Fig. 5b lower panels and Fig. 5c). Interestingly, among the group of lentiviral vectors expressing the miR-17∼92 cluster with one miRNA subfamily deleted, deletion of the miR-17 subfamily almost completely abrogated the ability of this cluster to rescue the pro- to pre-B transition defect of Mb1tKO cells (Fig. 5c and Supplementary Fig. 5). Conversely, lentiviral expression of miR-17 alone significantly restored the pro- to pre-B-cell transition (Fig. 5c and Supplementary Fig. 5). Deletion of miR-19 or miR-92 subfamily had a minor effect on the rescue and expression of miR-92 and miR-19 family had no effect or minor effect in this process, respectively. Therefore, we conclude that the miR-17 subfamily plays a central role in regulating the pro- to pre-B transition during early B-cell development, with additional support from the miR-19 and miR-92 subfamilies. Our functional dissection of the miR-17∼92 cluster reveals a shift of power among these co-expressed miRNAs in two sequential and closely linked stages of B-cell development: although miR-17 plays a central role in controlling pro- to pre-B transition, miR-19 is critical for regulating B-cell central tolerance at the immature B-cell stage.

### PTEN, Phlpp2 and Bim do not control early B-cell development

**Figure 6:**
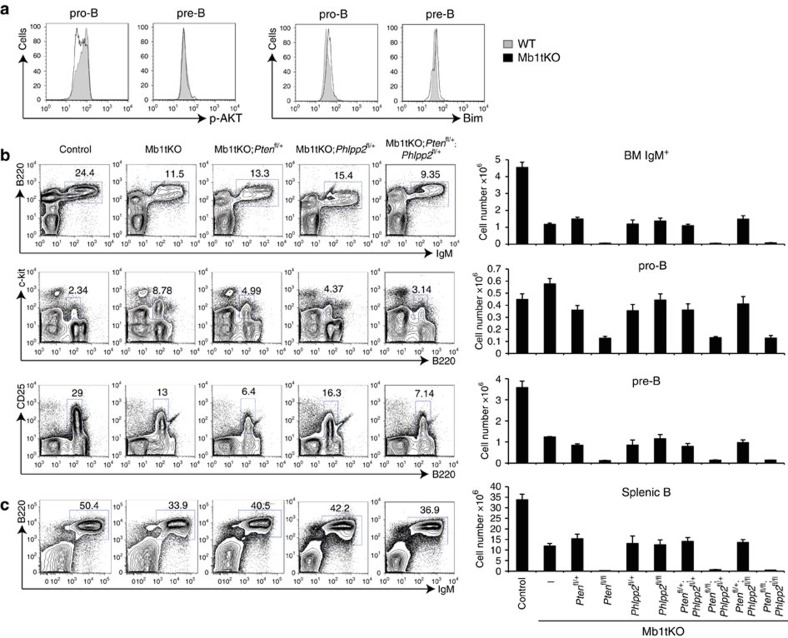
Ablation of *Pten* and *Phlpp2* do not rescue early B-cell development in mice deficient of the miR-17∼92 miRNA family. (**a**) Phospho-Akt T308 (p-AKT) and Bim protein levels were measured in pro-B and pre-B cells of control and Mb1tKO mice by flow cytometry and presented as overlay histograms. (**b**) Representative flow cytometry plots and cell numbers of IgM^+^ B cells (B220^+^IgM^+^), pro-B cells (B220^int^ckit^+^) and pre-B cells (B220^int^CD25^+^) in the bone marrow of mice of indicated genotypes. (**c**) Representative flow cytometry plots and cell numbers of splenic IgM^+^ B cells (B220^+^IgM^+^) in mice of indicated genotypes. Data are representative of 2 (**a**) or 8 (**b**,**c**), or pooled from 8 (**b**,**c**) independent experiments (mean±s.e.m. in **b**,**c**) with *n*=4 (**a**: Phospho-Akt(T308)) or 2 (**a**: Bim) in **a**, *n*=5 (Control, Mb1tKO, Mb1tKO;Pten^fl/+^Phlpp2^fl/+^ and Mb1tKO;Pten^fl/+^Phlpp2^fl/fl^), 7 (Mb1tKO;Pten^fl/fl^), 9 (Mb1tKO;Pten^fl/+^), 8 (Mb1tKO;Phlpp2^fl/fl^), 6 (Mb1tKO;Phlpp2^fl/+^ and Mb1tKO;Pten^fl/fl^Phlpp2^fl/+^) or 3 (Mb1tKO;Pten^fl/fl^Phlpp2^fl/fl^) in **b** (BM Ig+ and preB) and **c**, and *n*=4 (Control and Mb1tKO), 5 (Mb1tKO;Pten^fl/fl^, Mb1tKO;Pten^fl/+^Phlpp2^fl/+^, Mb1tKO;Pten^fl/+^Phlpp2^fl/fl^ and Mb1tKO;Pten^fl/fl^Phlpp2^fl/+^), 9 (Mb1tKO;Pten^fl/+^), 6 (Mb1tKO;Phlpp2^fl/fl^ and Mb1tKO;Phlpp2^fl/+^) or 3 (Mb1tKO;Pten^fl/fl^Phlpp2^fl/fl^) in **b** (proB).

The PI3K pathway has been shown to control early B-cell development^36^. Our results showed that the miR-17∼92 family miRNAs suppress the expression of PTEN and Phlpp2 in immature B cells (Fig. 3a) and mature B cells^25^. Moreover, flow cytometry analysis of pAkt in developing B cells of Mb1tKO mice showed decreased Akt phosphorylation in pro-B cells when compared with their WT counterparts (Fig. 6a). Previous studies have also suggested Bim as a key functional target mediating miR-17∼92 regulation of B-cell development^19^,^37^. We found slightly increased protein Bim levels in Mb1tKO pro-B cells compared with WT cells (Fig. 6a). For those reasons, we speculated that miR-17∼92 family miRNAs regulate early B-cell development by suppressing the expression of PTEN, Phlpp2 and Bim.

To test whether decreased PTEN and Phlpp2 expression rescues the B-cell development defect of Mb1tKO mice, we generated a panel of mouse strains by introducing *Pten* and/or *Phlpp2* deletion to Mb1tKO mice. As shown in Fig. 6b,c and Supplementary Fig. 6a,b, heterozygous and homozygous deletions of *Pten* and *Phlpp2*, either individually or in combination, were not able to rescue the pro- to pre-B transition block in Mb1tKO mice. Interestingly, homozygous deletion of *Pten* was detrimental to pro-B cells on the Mb1tKO background, as shown by the complete loss of B220^int^cKit^+^ pro-B cells when both *Pten* alleles were deleted.

Previous studies showed that Mb1-Cre-mediated deletion of Dicer led to a severe block of early B-cell development at the pro- to pre-B transition^37^, which is similar to but stronger than that caused by germline deletion of miR-17∼92 (ref. 19), or by the complete deletion of the miR-17∼92 family miRNAs specifically in the B-cell lineage (that is, Mb1tKO mice in this study). An Eμ-*Bcl-2* transgene (*Bcl2*Tg) was able to partially rescue the pro- to pre-B transition block caused by Dicer deletion^37^,^38^. We therefore tested whether the same *Bcl2*Tg was able to restore B-cell development in Mb1tKO mice. As shown in Supplementary Fig. 6c,d, although *Bcl2*Tg did restore the splenic B-cell number, probably by prolonging the survival of mature B cells in the periphery^39^, it did not have any measurable effect on the pro- to pre-B transition block in Mb1tKO mice. Taken together, these results exclude PTEN, Phlpp2 and Bim as major mediators of the regulation of early B-cell development by the miR-17∼92 family miRNAs. Thus, other target genes and molecular pathways must play more important roles in this process.

## Discussion

The present study revealed critical roles of the miR-17∼92 family miRNAs in two sequential events in the development of B cells: the pro- to pre-B cell transition and the establishment of B-cell central tolerance. The use of two novel *in vivo* models, Mb1tKO and the IgM^b^-macroself mice, enables us to dissect the dynamic functional contribution of individual members of a miRNA cluster in two closely linked developmental stages of B cells. As miR-17∼92 plays essential roles in both events, functional analysis of individual miRNAs encoded by this cluster in each event provides new insights into the question of specificity versus redundancy of individual members of heterogeneous miRNA clusters. Our findings demonstrate that different members of the miR-17∼92 cluster play central roles in controlling B-cell development and tolerance through different molecular pathways.

To our knowledge, miR-17∼92 is the first miRNA cluster that has been discovered to control B-cell tolerance. During early B-cell development, miR-17∼92 miRNAs are relatively abundant in pro- and pre-B cells, but their expression goes down drastically when developing B cells transit from pre-B to immature B cells^13^,^18^. As miR-17∼92 is generally thought to play pro-survival and pro-proliferation roles, keeping its expression level low renders immature B cells sensitive to self antigen-induced apoptosis, when the opportunity of receptor editing is exhausted. Among the six miRNAs encoded by miR-17∼92, miR-19 plays a key role in controlling B-cell central tolerance, at least partly through suppressing the expression of PTEN, a negative regulator of the PI3K-Akt pathway. This PI3K-Akt pathway was previously shown to be essential for BCR tonic signalling, which is essential for the survival of mature B cells^31^. Our results demonstrate that this pathway also plays critical roles in supporting the survival of immature B cells.

Several previous studies suggested that the miR-17∼92 family miRNAs are essential for the pro- to pre-B transition during early B-cell development, but discrepancy exists among those reports. In a study employing Mb1-Cre-mediated deletion of Dicer, which abolished the expression of all mature miRNAs, B-cell development was severely blocked at the pro- to pre-B transition. miR-17∼92-mediated suppression of Bim expression was proposed to be the major underlying mechanism^37^. Indeed, germline deletion of miR-17∼92 caused a partial B-cell development block at the pro- to pre-B transition and the Bim protein level was higher in miR-17∼92-deficient pre-B cells^19^. The pro- to pre-B transition block caused by Dicer deletion was partly rescued by a Eμ-*Bcl2* transgene, further supporting an important role of Bim in mediating miR-17∼92 control of early B-cell development^37^. However, another study reported that transgenic mice with ubiquitous overexpression of miR-17, a member of the miR-17∼92 cluster, exhibited a severe reduction in the number of B lineage cells in both the bone marrow and the spleen^34^. The latter study casted doubt on the cell autonomous function of the miR-17∼92 family miRNAs in controlling early B-cell development. In the present study, we generated and analysed mutant mice harbouring B-cell-specific deletion of the miR-17∼92 family miRNAs (Mb1tKO mice). These mutant mice exhibited a pro- to pre-B transition block that is similar to that caused by Mb1-Cre-mediated deletion of Dicer and germline deletion of miR-17∼92 (refs 19, 37). Therefore, the miR-17∼92 family miRNAs do play a cell-intrinsic role in controlling early B-cell development. Our functional dissection experiments revealed that, among the six miRNAs encoded by miR-17∼92, miR-17 plays a central role in controlling the pro- to pre-B-cell transition, whereas miR-19 and miR-92 probably synergize with miR-17 to exert this function. Surprisingly, although PTEN is an important mediator of the miR-17∼92 function in immature B (shown in this study) and mature B cells^25^, it does not seem to play critical roles in miR-17∼92 regulation of early B-cell development. In addition, our results excluded an important role of miR-17∼92 regulation of Bim in controlling early B-lymphocyte development, which was suggested by previous studies^19^,^37^. Thus, other unknown molecular pathways must mediate miR-17∼92 family miRNA control of the pro- to pre-B-cell transition. The elucidation of these pathways warrants future investigation.

Our experimental approach provided a unique opportunity to investigate how individual members of a heterogeneous miRNA cluster work together to exert their functions in a dynamic manner. Previous studies of miR-17∼92 highlighted the complexity of this issue. In a c-Myc-driven lymphomagenesis model and a Notch-driven leukemogenesis model, miR-19 was shown to be the key oncogenic member of the miR-17∼92 cluster^40^,^41^,^42^. A recent study reported that miR-92 negatively regulated the oncogenic cooperation between miR-19 and c-Myc, suggesting functional antagonism among members of this cluster^43^. In another study employing retroviral overexpression of miR-17∼92 and its individual members in HSCs, overexpression of miR-19a and miR-92a resulted in B-cell hyperplasia and erythroleukemia, respectively. Interestingly, miR-92a-induced erythroleukemia development was abrogated by co-overexpressing miR-17, suggesting functional antagonism between miR-17 and miR-92a^44^. Our study focused on functional dissection of the miR-17∼92 cluster at two closely linked developmental stages of the B-cell lineage. While miR-17 plays a central role in controlling the pro- to pre-B transition, miR-19 is critical for regulating B-cell central tolerance at the immature B-cell stage. In both processes, other miRNAs in this cluster play supporting, instead of antagonistic, roles (that is, miR-19 and miR-92 contribute to the regulation of pro- to pre-B transition, whereas miR-17 partly controls B-cell central tolerance). Therefore, there is a shift of power among members of the miR-17∼92 cluster at these two closely linked developmental stages of the B-cell lineage. It is conceivable that individual members of a heterogeneous miRNA cluster regulate different, yet overlapping, sets of target genes and might have different effects on the molecular pathways controlled by the cluster. It is also possible that at different developmental stages of a single cell lineage, or in different cellular contexts, the functional relevance of these molecular pathways may differ to a large degree. That would explain the differential contribution of individual members of a miRNA cluster to its roles in different cellular contexts. Thus, miR-19 plays a critical role in regulating B-cell central tolerance through suppressing the expression of *Pten*, whereas miR-17 controls the pro- to pre-B transition through other unknown molecular pathways.

In summary, our study identified critical roles of miR-17∼92 in two closely linked developmental stages of the B-cell lineage, pro- to pre-B transition and immature B cells. Interestingly, different members of this cluster play central roles in these two processes by regulating different molecular pathways. These findings illustrate dynamic functional specificity of individual members of a heterogeneous miRNA cluster in B-cell development.

## Methods

### Mice

The generation of miR-17∼92 Tg (Jax stock 008517), miR-17∼92^fl/fl^ (Jax stock 008458), miR-106a∼363^−/−^ (Jax stock 008461), miR-106b∼25^−/−^ (Jax stock 008460), CD19-Cre (Jax stock 006785), Mb1-Cre, IgM^b^-macroself, *Pten*^fl/fl^ (Jax stock 006440) and Eμ-*Bcl-2* transgenic mice was previously reported^19^,^24^,^26^,^35^,^38^,^45^,^46^. Phlpp2^fl/fl^ mice were generated in the Xiao lab using EUCOMM ES clone HEPD0619_1_C08. All these strains are in the C57BL/6J genetic background. Both male and female mice of 8–12 weeks of age were used for most experiments. For early B-cell development characterization, mice were analysed at 6–8 weeks of age. All mice were bred and housed under specific pathogen free (SPF) conditions. All animal experiments were approved by the Animal Care and Use Committee of The Scripps Research Institute.

### Lentiviral vector generation and virus production

Lentiviral plasmids pWPXLd, pMD2.G and psPAX2 were gifts from Dr Didier Trono laboratory (Addgene plasmids #12258, 12259 and 12260). The expression vectors for the FL miR-17∼92, miRNA subfamily deletion mutants and individual miRNA components were constructed by stepwise cloning of various combinations of individual miRNAs (precursor sequences with 30–50 nucleotide flanking regions) into pWPXLd. The Ametrine expression vectors were constructed by replacing the GFP cassette of pWPXLd with the IRES-Ametrine 1.1, a violet-excitable yellow-fluorescing GFP variant. The *Pten*-coding sequence was subsequently cloned into this vector for producing *Pten*-expressing lentiviruses. Recombinant lentivirus was produced in 293T cells by co-transfecting the expression vectors with packaging constructs pMD2.G and psPAX2 by the calcium phosphate method. The virus-containing supernatant was harvested 48 h after transfection, filtered and concentrated using PEG-it Virus Precipitation Solution (System Biosciences).

### Bone marrow transduction and reconstitution experiments

HSCs were purified by enrichment of Sca-1^+^ cells using magnetic cell separation followed by isolation of bone marrow side population cells using FACS based on the capacity of these cells to actively exclude the vital dye Hoechst 33342 (ref. 47). Briefly, bone marrow cells were prepared from the femurs and tibias. After red blood cell lysis, Sca-1^+^ cells were enriched by anti-Sca-1 MACS MicroBeads according to manufacturer's instruction (Miltenyi Biotec). Sca-1^+^ cells were re-suspended in HBSS containing 2% fetal bovine serum (FBS), penicillin–streptomycin and 10 mM Hepes buffer, and stained with 8.8 μg ml^−1^ Hoechst 33342 (Invitrogen) at 5 × 10^6^ cells per ml. After incubation at 37 °C for 90 min, the Hoechst 33342-negative side population cells were isolated using a FACSAria cell sorter (BD Biosciences).

Purified HSCs were re-suspended in StemSpan medium (StemCell Technologies) supplemented with 100 μg ml^−1^ stem cell factor (SCF), 50 μg ml^−1^ thrombopoietin (TPO), 100 μg ml^−1^ Fms-related tyrosine kinase 3 ligand (FTL3), 10 μg ml^−1^ interleukin-6 (all from PeproTech) and 2 μg ml^−1^ Polybrene (Sigma-Aldrich). Lentiviral transduction (multiplicity of infection=10) was performed in round bottom 96-well plates, using 10-15 × 10^3^ cells per 25 μl reaction volume, at 37 °C for 24 h.

Recipient mice were irradiated with two doses of 5 Gy, 3 h apart and subjected to bone marrow transplantation 2 h later by tail vein injection with: (i) 5 × 10^6^ bone marrow cells from the indicated donors or (ii) 10–15 × 10^3^ lentivirally transduced HSCs mixed with 1 × 10^6^ congenic, unfractionated bone marrow cells. Recipient mice were maintained with antibiotics-containing food or water for 30 days before switching to normal food and analysed at indicated time points.

### Antibodies, western blot and flow cytometry analysis

For western blotting, cells were lysed in RIPA buffer (140 mM NaCl, 10 mM Tris-HCl pH 8.0, 1 mM EDTA, 1% Triton X-100, 0.1% sodium deoxycholate and 0.1% SDS) supplemented with Halt Protease & Phosphatase Inhibitor Cocktail (Thermo Scientific). Cell lysates were resolved on 4–20% SDS–PAGE. Antibodies used for western blotting are anti-PTEN (Cell Signaling, 9559; dilution 1/1,000), anti-Phlpp2 (Bethyl, A300-661A-1; dilution 1/500), anti-Bim (Cell Signaling, 2933; dilution 1/1,000) and anti-β-actin (Sigma-Aldrich, AC-74; dilution 1/10,000). Images have been cropped for presentation. Full-size images are presented in Supplementary Fig. 7.

Cell surface staining and flow cytometry analysis were performed following established protocols. Intracellular staining was performed following fixation and permeabilization using BD Phosflow Perm Buffer II. Stained cells were analysed on FACSCalibur or LSR II (BD Biosciences). Data were analysed with FlowJo software (Tree Star). Antibodies and reagents with the following specificities were used for staining: anti-B220 (RA3-6B2, 103236; dilution 1/200), anti-CD45.1 (A20, 110730; dilution 1/200), anti-CD45.2 (104, 109820; dilution 1/200), anti-CD19 (6D5, 115508; dilution 1/200), anti-CD25 (PC61, 102008; dilution 1/200), anti-TCRβ (H57, 109228; dilution 1/200) and anti-light chain λ (RML-42, 407306; dilution 1/400) from BioLegend; Annexin V (88-8007-74; dilution 1/100), anti-cKit (ACK2, 17-1172-81; dilution 1/100), anti-IgD (11-26, 12-5993-82; dilution 1/500) and anti-CD93 (AA4.1, 17-5892-83; dilution 1/200) from eBioscience; anti-active Caspase 3 (550480; dilution 1/5), anti-CD19 (1D3, 550992; dilution 1/400), anti-CD43 (S7, 553271; dilution 1/100), anti-B220 (RA3-6B2, 553093; dilution 1/200) and anti-light chain κ (187.1, 561354; dilution 1/50) from BD Bioscience; anti-IgM (115-097-020 and 115-175-075; dilution 1/500) from Jackson ImmunoResarch; and anti-Bim (C34C5, 2933; dilution 1/200) and anti-phospho-Akt-Thr308 (244F9, 4056; dilution 1/100) from Cell Signaling.

### *In vitro* immature B-cell culture and enrichment

Total bone marrow cells were isolated from the femur and tibia. B lineage cells were enriched by magnetic cell sorting using anti-CD19 MACS MicroBeads (Miltenyi Biotech). CD19^+^ cells were cultured in Advanced DMEM-reduced serum medium (Gibco) supplemented with 10% FBS, 55 μM β-mercaptoethanol, penicillin–streptomycin, 4 mM glutamine and 5 ng ml^−1^ recombinant human IL-7 for 5 days. Expanded immature B cells were enriched by incubating with biotin anti-IgM antibodies (RMM-1, 406503; dilution 1/500; BioLegend) at 4 °C followed by magnetic cell sorting using Streptavidin MicroBeads (Miltenyi Biotech).

### Immunization and ELISA assay

Antigens for immunization were prepared by mixing NP_36_-CGG (Biosearch Technologies) dissolved in PBS and 10% KAl(SO4)_2_ at 1:1 ratio and adjusting pH to 7, to form precipitate. Five micrograms of NP-CGG precipitated in alum was injected intraperitoneally for NP-specific antibody responses.

Microtitre plates were coated with the following: (i) 10 μg ml^−1^ NP_30_-BSA (Biosearch Technologies) in PBS, for measurement of NP-specific antibody, or (ii) 2.5 μg ml^−1^ goat anti-mouse κ- and goat anti-mouse λ-antibodies diluted in PBS containing Ca^2+^ and Mg^2+^, pH 7.0–7.2, for measurement of total serum antibodies. Nonspecific binding was blocked with 0.5% BSA in PBS. Serum samples were serially diluted in 0.5% BSA in PBS and were incubated in blocked plates overnight at 4 °C. Plates were incubated for 2 h with biotin-conjugated anti-IgM (1020-08, 1 μg ml^−1^, Southern Biotech), anti-IgG1 (1070-08, 1 μg ml^−1^, Southern Biotech) or anti-IgG (1030-08, 1 μg ml^−1^, Southern Biotech), for 1 h with streptavidin–alkaline phosphatase (Roche), and then with alkaline phosphatase substrate solution containing 4-nitro-phenyl phosphate (Sigma) for colour development, followed by quantification on a VERSAmax microplate reader (Molecular Devices).

### Northern blotting

Total RNA was extracted from miRNA-encoding lentivirus-transduced HeLa cells using TRIzol Reagent (Life Technologies) following the manufacturer's instructions. Ten micrograms of total RNA was used to detect miRNAs. DNA oligonucleotides antisense to mature miRNAs were used as probes. U6 small nuclear RNA was used as internal control for normalization. Northern blotting results were acquired on a Typhoon 9410 imager (GE Healthcare) and analysed using the ImageQuant software.

### Luciferase reporter assays

The 3′-UTR fragments of *Pten* and *Phlpp2* containing predicted miR-19-binding sites (Supplementary Data set 1) were cloned into the psiCHECK2 vector (Promega), to generate *Pten* and *Phlpp2* reporter plasmids. The miR-19-binding site was subsequently mutated to generate the Pten–3′-UTR-mut construct. HeLa cells were plated into 24-well plates at 4 × 10^4^ cells per well 24 h before transfection with 0.1 μg reporter plasmid and 0.4 μg miR-19-expressing pWPXLd plasmid using the Lipofectamine 2000 transfection reagent (Invitrogen). Luciferase assays were performed 48 h after transfection using the Dual-Luciferase Reporter Assay System (Promega) following the manufacturer's protocol. The Rluc activity was normalized by the firefly luciferase activity (Luc) and expression is presented as Rluc/Luc ratio, which was arbitrarily set as 1 for the empty pWPXLd plasmid for each reporter.

### *IGHV* gene analysis

B cells from the spleen and lymph nodes were enriched by magnetic cell sorting using anti-CD19 MACS MicroBeads (Miltenyi Biotech). Purified B cells (0.5 × 10^6^ cells per plate) were cultured in the presence of irradiated 40LB feeder cells (3 × 10^6^ cells per plate) in 40 ml RPMI-1640 medium (Gibco) supplemented with 10% FBS, 1 mM soldium pyruvate, 55 μM b-mercaptoethanol, penicillin–streptomycin, 10 mM HEPES and 1 ng ml^−1^ recombinant IL-4 (PeproTech) and harvested after 5 days^48^.

Sample preparation and sequencing of mouse antibody libraries were performed based on a similar procedure that has been described previously^49^. Briefly, total RNA was extracted from 10 to 20 million *in vitro*-expanded B cells into 30 μl of water with RNeasy Mini Kit (Qiagen). For unbiased antibody repertoire analysis, 5′-rapid amplification of cloned/complementary DNA ends (RACE) was performed with SMARTer RACE cDNA Amplification Kit (Clontech). The immunoglobulin PCRs were set up with Platinum Taq High-Fidelity DNA Polymerase (Life Technologies) in a total volume of 50 μl, with 5 μl of cDNA as template, 1 μl of 5′-RACE primer and 1 μl of 10 μM reverse primer. The 5′-RACE primer contained a PGM P1 adaptor, whereas the reverse primer contained a PGM A adaptor. We adapted the 3′-Cγ1-3 inner primers and 3′-Cμ inner primers, 3′-mCκ outer primer and 3′-Cmλ outer primer as reverse primers for 5′-RACE PCR processing of the H, κ and λ chains, respectively^50^. Twenty-five cycles of PCRs were performed and the expected PCR products (500–600 bp) were gel purified (Qiagen). The antibody heavy (H) and light (κ and λ) chain libraries were quantified using Qubit 2.0 Fluorometer with Qubit dsDNA HS Assay Kit and then used at a ratio of 1:1:1 for all the PGM sequencing experiments. The dilution factor required for Ion Torrent PGM template preparation was determined such that the final concentration was 50 pM. Template preparation was performed with the Isothermal Amplification Kit obtained from the Early Access Program. Before PGM sequencing, quality control of the template was determined by the Qubit 2.0 Fluorometer with the Ion Sphere Quality Control Kit. Sequencing was performed on the Ion Torrent PGM sequencer with the PGM Hi-Q 400 Kit using an Ion 314 v2 chip for a total of 1,100 nucleotide flows. Raw data were processed without the 3′-end trimming in base calling, to extend the read length.

Bioinformatics analysis of antibody sequence data followed the human antibodyomics pipeline^49^,^51^,^52^,^53^,^54^,^55^. Specifically, the mouse H, κ and λ germline genes from IMGT (http://www.imgt.org) including the *V*, *D* and *J* segments were incorporated into the pipeline where such information is required for gene assignment (step 2), error correction (step 3) and determination of H/LCDR3 and variable region boundaries (step 5). For heavy chains, 313 VH genes along with 39 *DH* and 9 *JH* genes were compiled into three libraries, whereas for light chains, 151 *VK* genes along with 5 *JK* genes and 19 *VL* genes with 7 *JL* genes were used in library construction, respectively. The mouse antibodyomics pipeline consists of five consecutive steps. Given a data set of NGS-derived mouse antibody sequences, each sequence was (1) reformatted and labelled with a unique index number; (2) assigned to *V*, *D* (for heavy chain only) and *J* gene families using the current rhesus macaque germline gene database and an in-house implementation of IgBLAST, and sequences with E-value>10^−3^ for *V* gene assignment were removed from the data set; (3) subjected to a template-based error-correction procedure, in which insertion and deletion (indel) errors in the *V* gene segment were detected based on the alignment to their respective germline gene sequences. It is noteworthy that only indels of less than three nucleotides were corrected; (4) compared with the template antibody sequences at both the nucleotide level and the amino acid level using a global alignment module in CLUSTALW2 (ref. 56); (5) subjected to a multiple sequence alignment-based procedure to determine the complementarity determining region 3 (CDR3), which was further compared with the template CDR3 sequences at the nucleotide level and to determine the sequence boundary of the *V*(*D*)*J* coding region. After FL variable region sequences were obtained, a bioinformatics filter was applied to detect and remove erroneous sequences that may contain swapped gene segments due to PCR errors. Specifically, a FL read would be removed if the *V* gene alignment was <220 bp. The processed and annotated antibody chain sequences were then subjected to germline gene frequency analysis.

### Statistical analysis

Data were analysed using unpaired two-tailed Student's *t*-test (*P<0.05, **P<0.01 and ***P<0.001). Results are shown as mean with error bars indicating±s.e.m.

### Data availability

The authors declare that the data supporting the findings of this study are available within the article and its Supplementary Information files, or from the authors upon a reasonable request. The IGHV deep-sequencing data sets are available in SRA database with the accession code SRP075587.

## Additional information

**How to cite this article:** Lai, M. *et al*. Regulation of B-cell development and tolerance by different members of the miR-17∼92 family microRNAs. *Nat. Commun.* 7:12207 doi: 10.1038/ncomms12207 (2016).

## Supplementary Material

Supplementary Information Supplementary Figures 1 - 7

Supplementary Dataset 1 Pten and Phlpp2 3'UTRs, and cloned fragments containing miR-19-binding sites for reporter assays.
