# Supplementary material for: Regulation of B-cell development and tolerance by different members of the miR-17∼92 family microRNAs
Source: Nat Commun. 2016 Aug 2;7:12207. doi: 10.1038/ncomms12207 (PMC4974641; doi:10.1038/ncomms12207)
Supplement: Supplementary Information — Supplementary Figures 1 - 7 [file ncomms12207-s1.pdf]

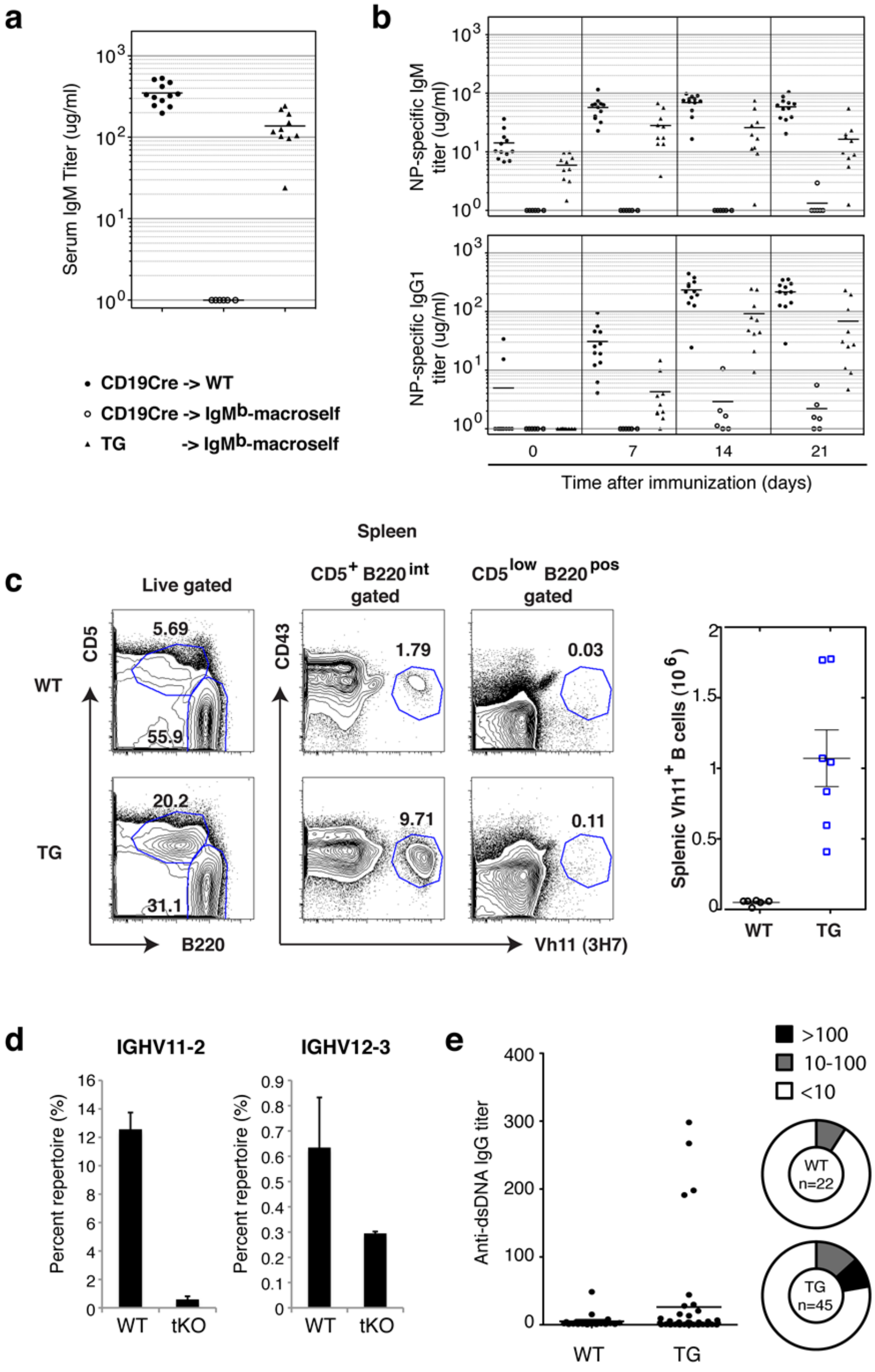


**Supplementary Figure 1 | Functional analysis of B cells in TG->IgM$^{b}$-macroself chimeras and autoreactive B cells in TG mice.**

(**a**) Total serum IgM concentrations in WT and IgM$^{b}$-macroself recipient mice were determined by ELISA at 2 months after bone marrow reconstitution. (**b**) Recipient mice in **a** were immunized with NP-CGG/alum. NP-specific IgM (upper panel) and IgG1 (lower panel) responses were assessed by NP-specific ELISA. Each symbol represents an individual mouse, and the horizontal bar indicates the mean. (**c**) $V_H11^+$ B cells were detected by flow cytometry in the spleen of WT and TG mice. (**d**) IGHV11-2 and IGHV12-3 gene usage in C57BL/6J (WT) and tKO (CD19tKO or CD19-Cre;miR-

17~92$^{fl/fl}$;miR-106a~363$^{-/-}$;miR-106b~25$^{-/-}$) mice was determined by BCR repertoire analysis of splenic B cells. B cells were activated *in vitro* to facilitate the analysis. (**e**) Enzyme-linked immunosorbent assay (ELISA) of serum titers of anti-double stranded DNA (anti-dsDNA) IgG antibody in WT and TG mice at terminal analysis. The serum titer of 4-month-old MRL-lpr/lpr mice was arbitrarily set as 1000. The frequencies of mice with high (>100, black), medium (10-100, grey) and low (<10, white) levels of anti-dsDNA antibodies in WT and TG groups are shown in the pie charts on the right. Data are representative of 3 (**c**) or 2 (**d**) or pooled from 2 (**a,b,e**) or 3 (**c**) independent experiments (mean ± SEM in **d**) with n=12 (Cre to WT), 6 (Cre to IgM$^b$-macroself) or 10 (TG to IgM$^b$-macroself) in **a,b**, n=6 (WT) or 7 (TG) in **c**, n=2 (WT and tKO) in **d**, and n=22 (WT) or 45 (TG) in **e**.

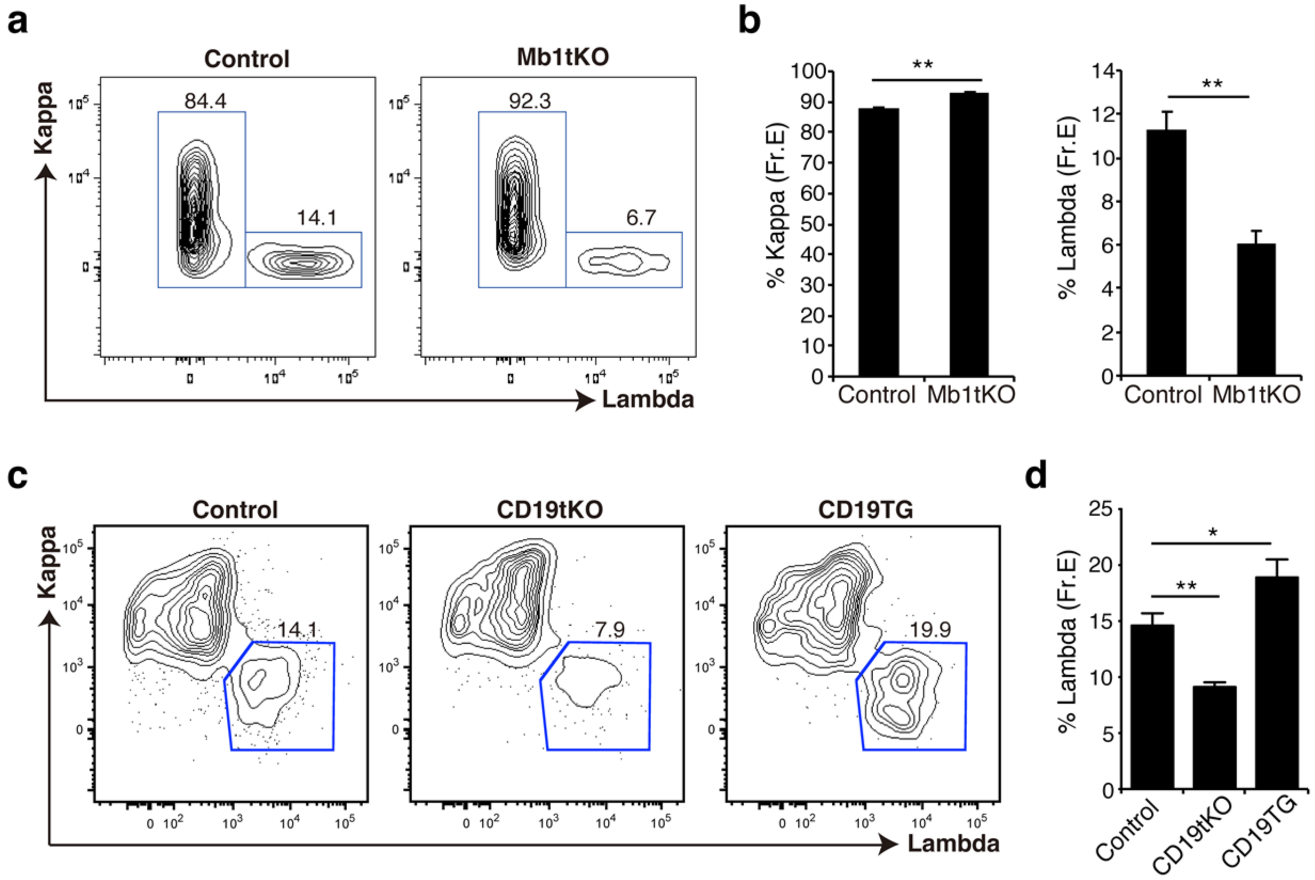


**Supplementary Figure 2 | miR-17~92 regulates receptor editing.**
(**a**) Representative flow cytometry plots showing kappa- and lambda-positive cells in immature B cells in the bone marrow (Fraction E) of control and Mb1tKO mice. (**b**) Percentages of Kappa- and lambda-positive immature B cells in the mice analyzed in **a**. (**c**) Representative FACS plots of kappa- and lambda-positive cells in immature B cells in the bone marrow of control, CD19tKO and TG (CD19TG) mice. (**d**) Percentages of lambda-positive immature B cells in the mice analyzed in **c**. *<0.05, **P<0.01 (two-tailed Student's t test). Data are representative of 2 (**a**) or 5 (**b**) or pooled from 2 (**a**) or 5 (**b**) independent experiments (mean ± SEM in **a,b**) with n=12 (Control and Mb1tKO) in **a**, and n=7 (Control), 10 (tKO) or 5 (TG) in **b**.

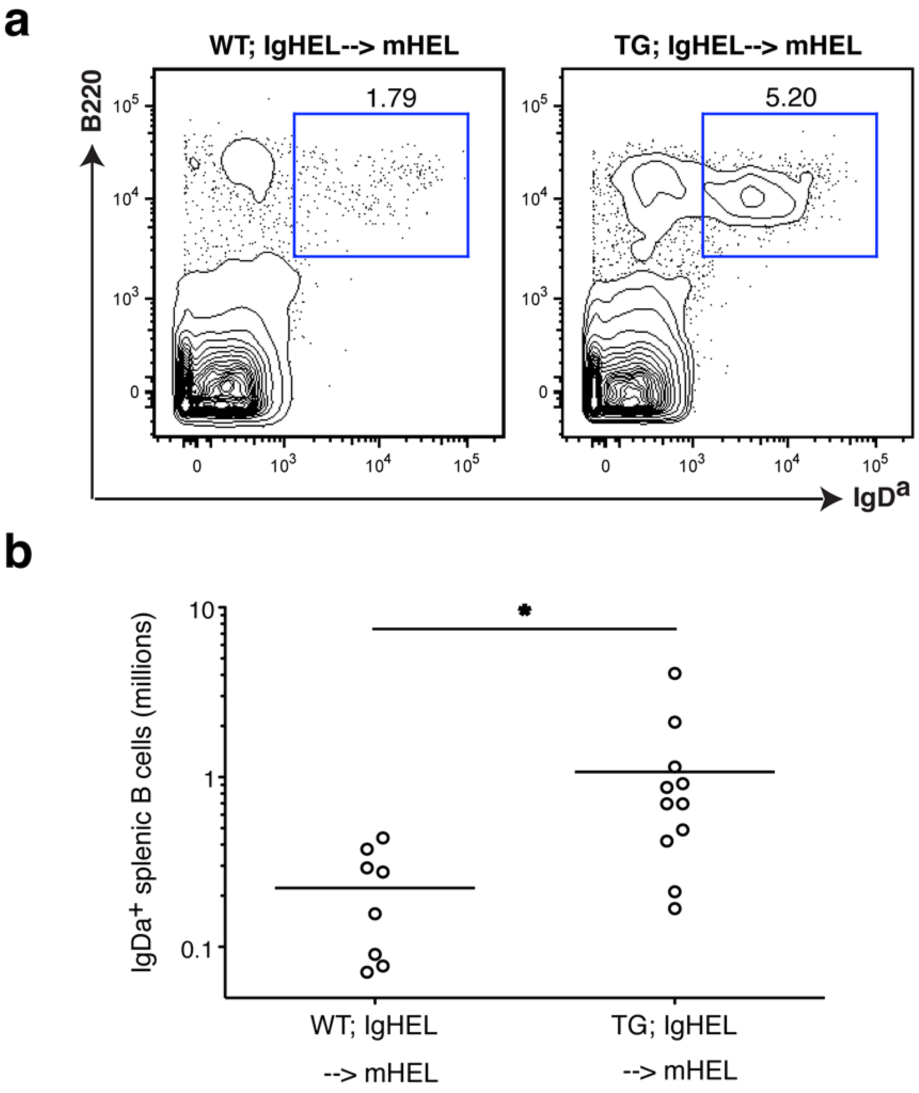


**Supplementary Figure 3 | miR-17~92 regulates B cell central tolerance in the IgHEL:mHEL model.**

(**a**) Representative flow cytometry plots showing splenic IgHEL (IgDa$^+$) B cells of mHEL recipient mice reconstituted with bone marrow cells from WT;IgHEL or TG;IgHEL mice. Recipient mice were analyzed at 8 weeks after reconstitution. (**b**) Splenic IgHEL (IgDa$^+$) B cell numbers in the mice analyzed in **a**. *<0.05 (two-tailed Student's t test). Data are representative of (**a**) or pooled from (**b**) 3 independent experiments with n=8 (WT) or 11 (TG).

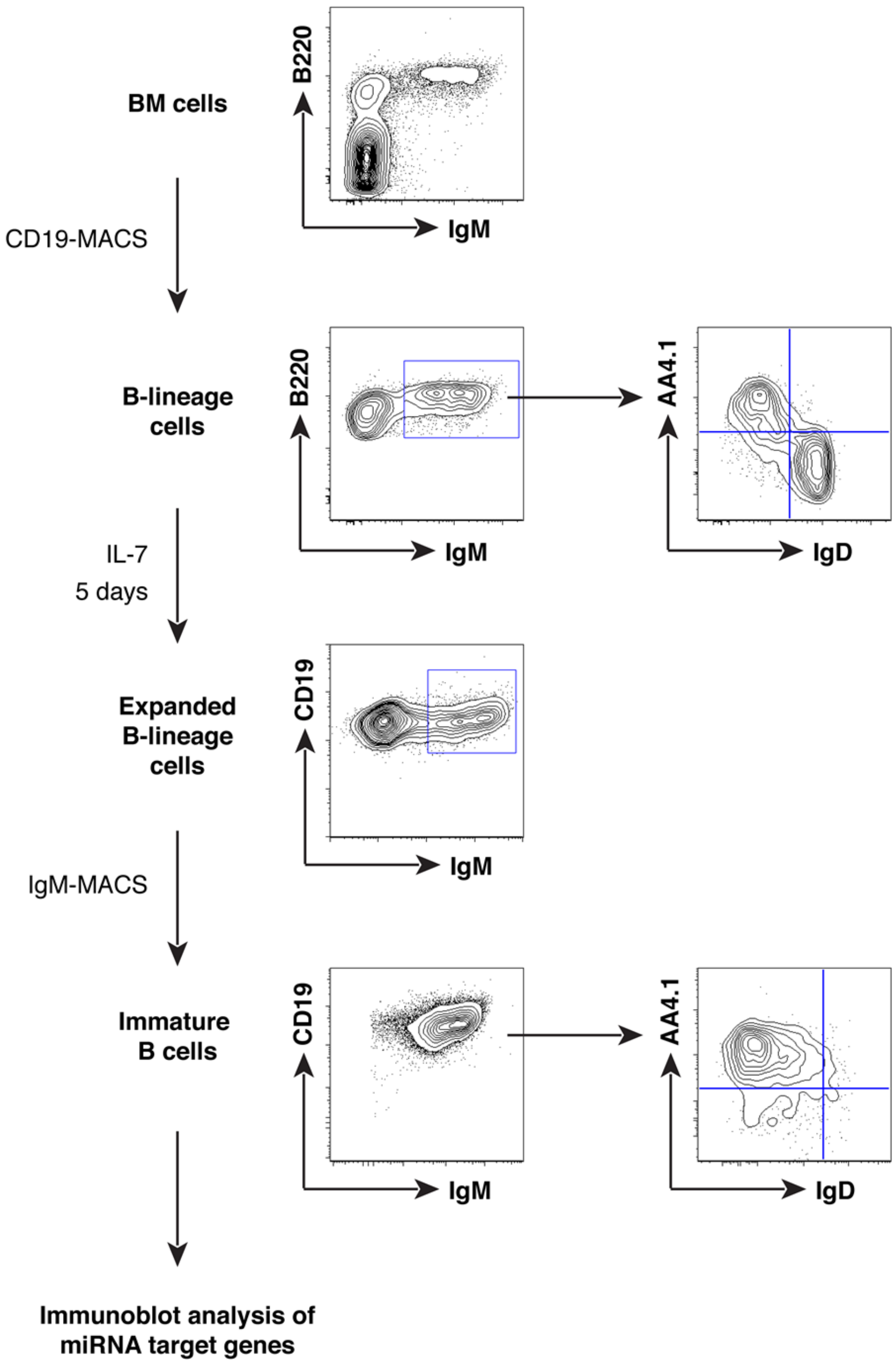


**Supplementary Figure 4 | *In vitro* culture and enrichment of immature B cells for the detection of miR-17~92 target gene expression.**
Representative flow cytometry plots of the cultured cells before and after MACS purification, showing the percentage and purity of immature B cells with a $CD19^{+}IgM^{+}AA4.1^{+}IgD^{-}$ surface phenotype. Data are representative of 3 independent experiments.

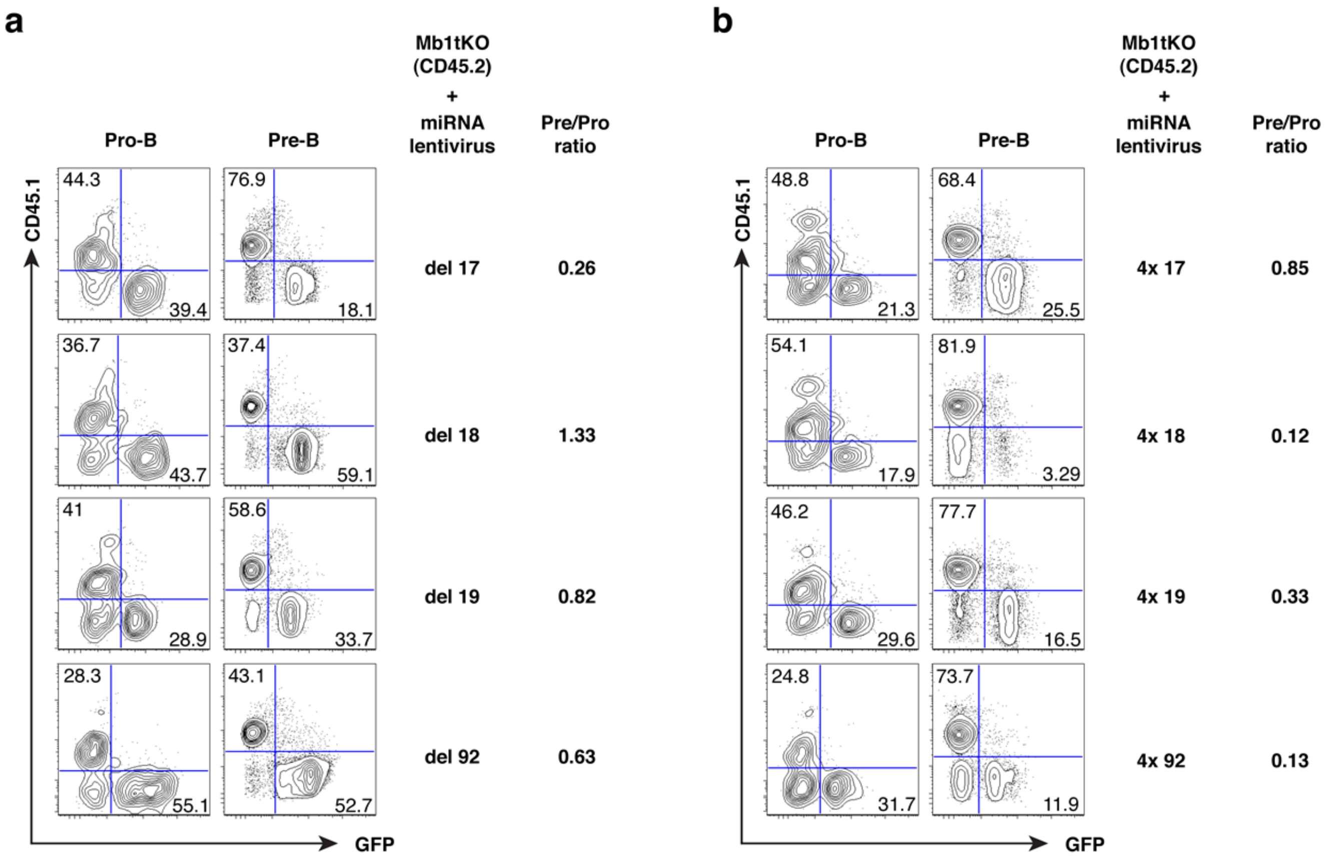


**Supplementary Figure 5 | miR-17 plays a central role in regulating the pro-B to pre-B transition.** Representative flow cytometry plots of pro- and pre-B cells of the mixed bone marrow chimeras, showing contributions from the WT-derived cells ($CD45.1^+$) and the lentivirus-transduced cells ($GFP^+$). HSCs from the Mb1tKO were transduced with lentivirus expressing (**a**) subfamily deletions, or (**b**) individual miRNAs of the miR-17~92 cluster, and mixed with BM cells from the congenic WT mice to generate the mixed bone marrow chimeras. Data are representative of 6 independent experiments with n=7 (del17) or 4 (del18, 19, 92) in **a**, and n=8 (4x17), 6 (4x18 and 4x92) or 7 (4x19) in **b**.

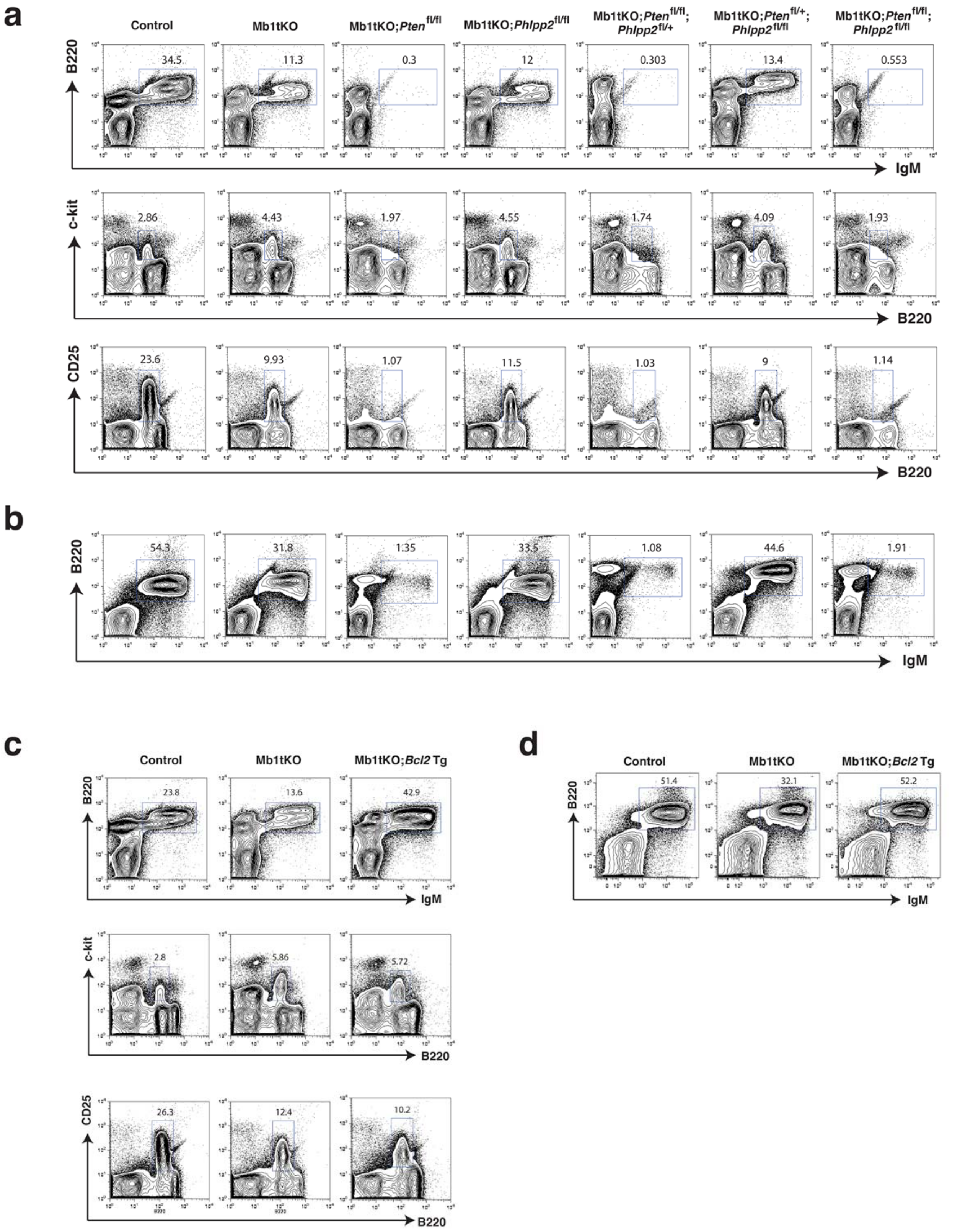


**Supplementary Figure 6 | *Pten* and *Phlpp2* ablation or transgenic *Bcl2* expression does not rescue early B cell development in mice deficient of the miR-17~92 miRNA family.**

Representative flow cytometry plots of (**a,c**) bone marrow IgM$^+$ B cells (B220$^+$IgM$^+$), pro-B cells (B220$^{int}$ckit$^+$) and pre-B cells (B220$^{int}$CD25$^+$), and (**b,d**) splenic IgM$^+$ B cells (B220$^+$IgM$^+$) in mice of

indicated genotypes. Data are representative of 8 (**a,b**) or 2 (**c,d**) independent experiments with n=5 (Control, Mb1tKO and Mb1tKO;Pten$^{fl/+}$Phlpp2$^{fl/fl}$), 7 (Mb1tKO;Pten$^{fl/fl}$), 8 (Mb1tKO;Phlpp2$^{fl/fl}$), 6 (Mb1tKO;Pten$^{fl/fl}$Phlpp2$^{fl/+}$) or 3 (Mb1tKO;Pten$^{fl/fl}$Phlpp2$^{fl/fl}$) in **a** (IgM-B220 and CD25-B220) and **b**, n=4 (Control and Mb1tKO), 5 (Mb1tKO;Pten$^{fl/fl}$, Mb1tKO;Pten$^{fl/+}$Phlpp2$^{fl/fl}$ and Mb1tKO;Pten$^{fl/fl}$Phlpp2$^{fl/+}$), 6 (Mb1tKO;Phlpp2$^{fl/fl}$) or 3 (Mb1tKO;Pten$^{fl/fl}$Phlpp2$^{fl/fl}$) in **a** (c-kit-B220), and n=5 (Control, Mb1tKO and Mb1tKO;Bcl2Tg) in **c,d**.

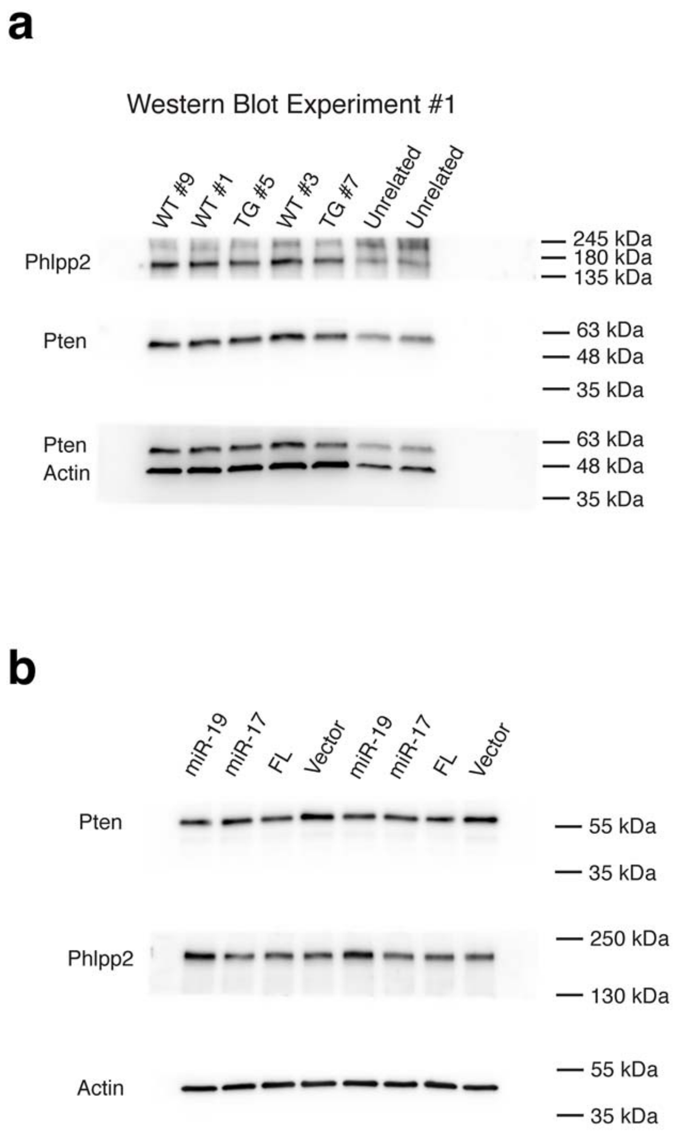


**Supplementary Figure 7 | Full-size scans of all Western blots shown in figures, with labeled lanes and molecular weight markers.**
Full size scan of the Western blots corresponding to Figure 3a (**a**) and Figure 3c (**b**) indicating the molecular weights of Pten, Phlpp2, Bim and actin.
