# Supplementary material for: Regulation of B-cell development and tolerance by different members of the miR-17∼92 family microRNAs
Source: Nat Commun. 2016 Aug 2;7:12207. doi: 10.1038/ncomms12207 (PMC4974641; doi:10.1038/ncomms12207)
Supplement: Supplementary Dataset 1 — Pten and Phlpp2 3'UTRs, and cloned fragments containing miR-19-binding sites for reporter assays. [file ncomms12207-s2.docx]

**Supplementary Dataset 1.** Pten and Phlpp2 3’UTRs, and cloned fragments containing miR-19-binding sites for reporter assays.

**1. Pten 3’UTR**

TTTTTTTTTTCTTATCAAGAGGGATAAAATACCATGAAAAAAAAAAAACTTGAATAAACTGAAATGGACCTTTTTTTTTTTTTTTTTTTTTTAAATGGCAATAGGACATTGTGTCAGATTGCAGTTATAGGAACAATTCTCTTCTCCTGACCAATCTTGTTTTACCCTATACATCCACAGGGTTTTGACACTTGTTGTCCAGTTAAAAAAAGGTTGTGTAGCTGTGTCATGTATATACCTTTTTGTGTCAAAAGGACATTTAAAATTCAATTAGGATAAATAAAAGATGGCACTTTCCCATTTTATTCCAGTTTTATAAAAAGTGGAGACAGGCTGATGTGTATACGCAGGAGTTTTTCCTTTATTTTCTGTCACCAGCTGAAGTGGCTGAAGAGCTCTGATTCCCGGGTTCACGTCCTACCCCTTTGCACTTGTGGCAACAGATAAGTTTGCAGTTGGCTAAGGAAGTTTCTGCAGGGTTTTGTTAGATTCTAATGCATGCACTTGGGTTGGGAATGGAGGGAATGCTCAGAAAGGAATGTTTCTACCTGGGCTCTGGACCATACACCATCTCCAGCTCCTTAGATGCACCTTTCTTTAGCATGCTCCACTTACTAATCTGGACATCCGAGAGATTGGCTGCTGTCCTGCTGTTTGTTTGTGCATTTTAAAGAGCATATTGGTGCTAGACAAGGCAGCTAGAGTGAGTATATTTGTAGTGGGGTACAGGAATGAACCATCTACAGCATCTTAAGAATCCACAAAGGAAGGGATATAAAAAAAGTGGTCATAGATAGATAAAAGACACAGCAGCAATGACTTAACCATACAAATGTGGAGGCTTTCAACAAAGGATGGGCTGGAAACAGAAAATTTGACAATGATTTATTCAGTATGCTTTCTCAGTTGTAATGACTGCTCCATCTCCTATGTAATCAAGGCCAGTGCTAAGAGTCAGATGCTATTAGTCCCTACATCAGTCAACACCTTACCTTTATTTTTATTAATTTTCAATCATATACCTACTGTGGATGCTTCATGTGCTGGCTGCCAGTTTGTTTTTCTCCTTAAATATTTTATAATTCTTCACAGGAAATTTCAACTTGAGATTCAACAGTAAGCAGGTTTTGTTTTTTTTTTTTCCTAGAGATTGATGATGCGCGTCCTCAGTCCAGTGGCTGTCAGACGTTCAGCCCCTTTGACCTTACACATTCTATTACAATGAGTTTTGCAGTTTTGCACATTTTTTTTAAATGTCATTAACTGTTAGGGAATTTTACTTGAATACTGAATACATATAATGTGTATATTAAAAAAGTCATTGTTTGTGTTAAAAAAGAAATTAGAGTTGCAGTAAATTTACAGCACTGCACGAATAATAAGGCATTGAAGTTTTTCAGTAGAAATTGTCCTACAGATGCTTTATCGACTTGCTATTGGAAGAATAGATCTTCTTAAATGTGCAGTGTTGAGTCACTTCGTTATAGTGGTAGAGTTGGGATTAGGGCTTCAATTTTACTTCTTAAATATCATTCTATGTTTGATATGCCCAGACTGCATACAATTTAAAGCAAGAGTACAACTACTATCGTAATGGTAATGTGAAGATGCTATTACAAAGGATCTCCTCCCAACCCCTCGGGAATTTGGTGTCTTTCAAATTATATCTTGACCTTGACATTTGAATATCCAGCCATTATTAGATTTCTTAATGGTGTGAAGTCCCATTTTCAATAACTTATTGGTGCTGAAATTGTTCACTAGCTGTGGTCTGACCTAGTTAATTTACAAGTACAGATTGCATAGGACCCACTAGAGAAGCATTTATAGTTTGATGGTAAGTAGATTAGGCAGAACGCCATCTAAAATATTCTTAGAAAATAATGTTGATGTATTTTCCATACCTCATCAGTTTCACTCAACCAATAAAGTTTTTAAAATTGTAACAAAGCTCTTAGGATTTACACATTTATATTTAAACATTGATACATGAATATTGACTGACTGTTGATAAAGTCAGAGACAACTTTTCCTGAGATCTCACCATGGAAATCTGTACACCCCCTTGTCTTTCCTAAAAGCTGAAAGTGGCTGACTAAAATGCAAAGCAGCTGTTGATGTTTTGAAGATAGTGATAAACACTGTTCTTTGTTAGTTTTGGGCACAGCATGCTAAACTATAACTTGTATTGTTCCAATATGTAACACAGAGGGCCAGGTCATGAATAATGACATTACAATGGGCTGTTGCACTGTTAATATTTTTCCTTTGGAATGTGAAGGTCTGAATGAGGGTTTTGATTTTGAATGTTTCAGTGTTTTTGAGAAGCCTTGCTTACATTTTATGGTGTAGTCATTGGAAATGGAAAAATGGCATTATATATATATTATATATATATAAATATATATATTATACATACTCTCCTTACTTTATTTCAGTTACCATCCCCATAGAATTTGACAAGAATTGCTATGACTGAAAGGGTTTTGAGTCCTAATTCAAACTTTCTTTATGACAGTATTCACGATTAGCCTGAAGTGCATTCTGTAGGTGATCTCTCCCGTGTTTCTGGAATGCTTTCTTAGACTCTTGGATGTGCAGCAGCTTATGTGTCTGAAATGACTTGAAGGCATCACCTTTAAGAAGGCTTACAGTTGGGCCCCGTACATCCCAAGTCCTCTGTAATTCCTCTTGGACATTTTTGCCATAATTGTAAAAGGGTAGTTGAATTAAATAGCGTCACCATTCTTTGCTGTGGCACAGGTTATAAACTTAAGTGGAGTTTACCGGCAGCATCAAATGTTTCAGCTTTAAAAATAAAAGTAGGTTACAAGTTACATGTTTAGTTTTAGAAAATTTGTGCAATATGTTCATAACGATGGCTGTGGTTGCCACAAAGTGCCTCGTTTACCTTTAAATACTGTTAATGTGTCGTGCATGCAGACGGAAGGGGTGGATCTGTGCACTAAACGGGGGGCTTTTACTCTAGTATTCGGCAGAGTTGCCTTCTACCTGCCAGCTCAAAAGTTCGATCTGTTTTCATATAGAATATATATACTAAAACCATCCAGTCTGTAAAACAGCCTTACCCCGATTCAGCCTCTTCAGATACTCTTGTGCTGTGCAGCAGTGGCTCTGTGTGTAAATGCTATGCACTGAGGATACACAAATATGACGTGTACAGGATAATGCCTCATACCAATCAGATGTCCATTTGTTACTGTGTTTGTTAACAACCCTTTATCTCTTAGTGTTATAAACTCCACTTAAAACTGATTAAAGTCTCATTCTTGTCATTGTGTGGGTGTTTTATTAAATGAGAGTATTTATAATTCAAATTGCTTAAATCCATTAAAATGTTCAGTAATGGGCAGCCACATATGATTACAAAGTTCCTGTGCATTTTTCTATTTTTCCCCCTCCTTGCTATCCTTCCAAGCAAAGCATCTTTCTGTCATCTTGGTAGACACATACCTGTCTACTCATGGTTAAGAAGAGCACTTTAAGCCTTAGTCATCACTTAATAAGTTATTCCAGGCACAGTAAAAAGTTCAAGGTTCTTGGAAAACGGTGCTTATTTCTCTTCTTATAAGCCAGATGTCTGAAGATAGCCCTAACCCCAAGAACGGGCTTGATGTCTCAGGTCTGTTCTGTGGCTTTCTGTTTTTTTTAACACTGCAGTTGGCCATCAGCACATGGGAGGTTTCATCGGGACTTGTCCAGAGTAGTAGGCTCAAATATACTATCTCCTTTCTAATATTCTTAAAGGCTAAGGAGTCCTTTCAATATAACAGTAAGATAACTTGTGATGTTTTAGAAGTAAGCAGACCATTAATGTCAATGTGGAGTCTTAATGTTACATGAAGTTGATAGTTTCTCTGTGACCCATTTAAAAATACAAACCGAGTAGCATGCAATTATGTAAAGAAATATGAAGATTATATGTAGTCACACATTTTCTTTAGAATTCTTAGTTTGGTGAAAACTTGAATATAAAGGTATTTTGATTTATATGACATTTTGATGATATTTGAAAAAAAGGAATTTCCTGACATTTTGCTTTTAGATCATGTCCCCCATTGTGCTGTAATTTAAGCCAACTTGGTTCAGTGAATGCCATCACCATTTCCATTGAGAATTTAAAACTCACCAGTGTTTAACATGCAGGCTTCTGAGGGCTCCCGGAGAATCAGACCTTAAGCCCAGTTGATTTACTTCTAACGTGAAACTTCGAGTTCCTGTATACTTTGCTAGATAATTTGTGGTACATCTAAAGCTTAGTCTTAAGTGGCTTGTGTGTGGATTTTATTCAACATTCTTGTTGCTAGGGTAGAGAGAAATGTTGCTGAGTAGAAACAAGAGTACCCAGTTCAATGTGGTACAGAGAGCAGTCCCTAAAATCTGTACACAGTGTAATGGACCACTTTAGGAGTCAAGAGGCTGATTTTTCCTATGAAATTACATTGCAACAGGAAGCCTTCTAGTATAGTTCCTTTTACTGTTAGAATATGTTTTTATGCATACGCTATAGCTGCTTTCCCATCTTCCAACAACAGGTATCAGGATGTAAGCAAGCTTTAAACAGTGTGAAGATGGCAGGATAGTGTCATCGGTAACAGTCCTCTGACTCTAAATGTAGTTGCTCTGTAACACTTTGTGAATATAACATCACAATTCTCATGTCCTTGGGGGGGGGGGGCATACCCAGTATTAGTATGTTTTAGTGACTAAGCAATCATTTTTCTGTTTACTCATGTACATTTTCTCTTTAAAACTAAAACCTGTACTGTGTATGTCTCCAAAGCCTTTTAGCTTAGTTTTTAGGAAATGAACACTGAATGGATCACTTTTTAGTGTAGCAGGTATGGGATATGTGCATTATAGAGAGACCTTGTCAGCTCTCTGGGCCTATTTGAATGTTTATTGTTGGTGTGAGGATGGTAGGGGAATCAGTAAATACAAGTTACGTTGGTTTAGCAGAGCAAGCTCAGTGTGGGTATTTCTCTTTGAAGCGTGGTGCGTGACGCACTGTGAGTAGAGAATTTGGTCACCCTTTGAGTCCTCTTGCATTTTGCAAACTTGCTCAGCAAATGCGTACCTACCTTGCCCCCTAGGTAAAAGCAGGAACTACTACTGATTTATCTGTCACTCAGCTGTCTTTATATGTGTGCTTCTGTGACTTGTATCACACAAGAATCTTAAAGATTTCACAAATTGTTACCTTTTAGCTCTGAATGTTGAGTATTCTGGTGGGCTAACAACAAGACAAACTCTTGACAGTCATTTGAGAATTTTCATGAAACATTTAGCTGAAAACATTTTATAATTTATGAAAAAAATGTGTTACCTTAAACTTTTACATATGTGGGAGACATTAACTGCCATATTTGAGCATACTGAATTTTAAATTTAAAATAAAGCTGCATATTTTTAAATGAAATGTTTAACAAGGATTCATATTTTTTGTTTTTTAAGATTAAAAATAATTTATGTCTTCTCATGTGGAACCTCATCTGTCACAATGGTTAGATTATACAGAATGGAGCAAGGCTTGTAGTGGTTTAGCTTACAGTAAAATTCTTAATGTTTAGATGTGTTTACTTACTGGCTGTTATGTATACTTTTGAGATTTTCCACCTGTTCTGTGTAGTTTTCTAAATGATACTCCTACTTAAAAACAGCATTTTAGTATCTATTTTCTGTCTCCATTAAATGGTCCTCATTTTCTATTGAGTTTGGAAGTGTGCACATTGTGTGTGTGTGTGTGTGTGTGTGTGTGCACACGTGTGCGCGCCCGTGCGTGTGTCTATTTGTGGAGTTTGTATGGGAGAATTAGTTTTGAAAGTGCTAGAATAGAGATGAAATTTGGTTCAAGTAAAATTTTCCCACTGGGATTTTACAGTTTATTGTAATAAAATGTTAATTTTGGATGACCTTGAATATTAATGAATTTGTTAGCCTCTTGATGTGTGCATTAATGAGATATATCAAAGTTGTATATTAAACCAAAGTTGGAGTTGTGGAAGTGTTTTTATGAAGTTCCGTTTGGCTACCAATGGACATAAGACTAGAAATACCTTCCTGTGGAGAATATTTTTCCTTTAAACAATTAAAAAGGTTCATTATTTTTGA

(**1**) Pten 3’UTR fragments cloned for reporter assay

**Fragment Start End**

Pten 96 1512

(**2**) Predicted miRNA binding sites

**miRNA site Position Sequence Mutated sequence**

miR-19 1236-1243 TTTGCACA TATCCTCA

**2. Phlpp2 3’UTR**

TTGTACCTCTCGTGTGGACGCAGCCTGAGGGAGGGCTGTGTGGTATTGGGGAAGGGACCTTCAGGAGATATTTGCCTCAGTGTTTCAAACCATAGATAATATGGAGTAGAATTTGGAGCCAAAAAGTTGAGCACTAGCAGGGTCTGGCTCTTGATGGGCTCGGACCGCCATCAGTGTTAAGCTTCACATTGGACCTTGGTTGGACTCCCAGCGTTGCTGGGAGACAGCAGATGTTCTGTGTCAGTCCTGCCACCTGCCATTAACCCTTTCTCTCCTAGGATCATTTGAGAAATTGCCTGCCTGGGCAGGAAAGGGACTATTTCTGTGGAGGAAGTAACTGAAGGTTGATTCCCTTTACTAATTGCTGCTGATGGATCTCTGTGACAAGAGAAATCACCTTATCTCTCAGACTAACTAGTGGGATGTGATGTGACTAGTCACATGGCTTTTTATTCTCTAGGAGAATACAGCCTATCAAATGTCTACTGGAGATACAGAACCAATCAAGCAGGTGACTTATGTATGTAATGTTGTGTAACGAGAGCACTTTTCATTGACTGTGAACCTTTTATTTTTGAATCTGCACTCGGAGCCAATCTTCTTAGAGGCAGCCCAGCTCCTTTGTCCCTAGGTAGAAGTCATTGAGCATTGGAGCCTCTTTGAGGGTGCTTGGGAGGGTCCTGGAAGTTGATGTGCTGGATGTCAGACTGGGAAAGCTCCTGAGAAACTTGGGGTGGGTGGGTGGGGATTTATAAGATGGGGGAGGAGTGAGAACTAAGTCAAGTCAAAGAGACTCAGCCTTGGATAAGCTGACCCCTGCTAGGAACTAGGATCCCAAGGCAGGGACTTGTGGGTTTGCCTCTTTTCCTTAATGTGGTAGACTTGGCAGCTAGCCAGCCTCTTGACAGGTGGGCTGACTCCTTGAATTATAGCAAGTGAGTACAACAGGAGACCAGCTAGGAACATATATGGGCAGCCAGATCTCACAAGTTAGACTTGCTCCCCTCTCTCTCTCTCTCTCTCTCTCTCTCTTCTTACTCTTCTTTCTCTTCTCTTCTCTTCCCTCCTTCTCTTCCCTCCTTCCCTCCCTTCCTCCTTCCCTCCCTCCCTCTCTCAGGATGTTTTCTAGAATGTTGTAGTGAGTGTGTTGTTTGGCACTGTGCCCTTCTGAATATTGCTGAGTCCCCTGACATGGTGACATGGTTCAAAGTGCTGGCCTCAACACAGAGGAAGAAAGACTATTCAACTGAGCGATTGCTAAACTTACAGAAAACCTGTAATTTTTTTTTCACATTCCTCTTAACTCTAGCAAGTGACCACTTCTTGTGTATTTCTGTTGCTCTTTCCGTAGGTTACTAAGGAAACTCATTCCTATTTATTGATGTAAATTTTGGCATTGCTTGATTTTACTCAGTTGAAAAATGTGCTTTGAATTTTTAGAATATTGCACAACTAAAAAAATAGATTGGGGCATTTTTAAAAATTTTCCTTTATAGAAACAAATTGTTAAATATAAGATTGAAAGTGTCTATTTTTCCTTTGTTTTTGAGGAGGAAGTTTTTGTTTAAAGCAATACTGAAAAGCAGACAGATAATTCTGTCCTCAGAGGCCCTGTGCTCATGTGCACAGGGTTAGTGGTTACACTGGGGGTGGGGTGGGGTGGAGGATGCTCTATCACTGCAGCAGCTGCCTAATAATTCAGTTCTCAAGAATCTGAACGTGAGCCCCCAAGTCGGCAACACTCAGCTTCCTTCTGTCAGTTACTGTCAGTTACTGGGAAACATTAGAACTCAGGTGCTTGATTTGAGATGGGGACCCAGCGGCTTATACAGATTGTAGGTGTACTGCAGGGAGGAGAGACCATGGCACCACCTTGTGTTCCCACCTTATTTTTATATGGGAAACTTGAAGAAAAAAAAAAGAAAGAAAATGAGAGTGAAGGAAAAACACAAGGTGAGGATGCTGCAGAGAGCCTCCTGCTCAGCAGAGCCTTTGAGCTGAAGAACGAGAGGTGGTGATTATGAGAAACCAGTTATGTGGAAAAGCAGCACTTCGGTAGTCTTAGAACAATTGTCTTTTTATTATATGTGATCTGTTTTCTTATCCTCTGTTTGTTTACCTCCCCATTCACTAAAGTCGCCTCAAAACTCAGTTCTAAACGGATCTTGACAAACATGAACGTACTTCCCTTCTGGTTAGCAGAACTCTGGGGTGCAGGGTCCTTTGTGGGGGTTCAGGGTTCCTTAGGTTGTGTTGAGAAACCTCAGTGTAGGCCAAACCAAAATGGATGGTTCTGGTTTTGTAATTGTCTTTCTTTCATGTCCTATTATACTCTGTATTAAAAAACAGAGGGTTAAAAGGCCATAGTCCATTTCCCAAAACGTAATACACGCCTCTTGTAACAGTGAGGTGATTTGCTGATGTTTTTATCTGAGTAGGCTGTCAGGTGAGTGTGATTCCTTTCCCGTACCCAGAGGGTACATTCTCCAGGTGGCCAGAGGACAGGTTCCTTCTTACACAGAGCCTGCCCTCCCTCTATATGATTCTTGGTCTCTGCCCACATCTCCCGTTCAGTTAATTTAAACAAATCTCTGAGGCCAGGTGTGGTGGCACAGTGGCTCACACCTGCAAATCTGCACGCACATGGGAGGTTAAAGCAGGAGAATCAGGGATGAGGGTTAGCCTTGGCTACAGAGAGTTCAAATCCAGTCTGGTATACATGAGTCCCAGCCTCAAAACCAAACAAACAGGATTTCTGAAGAAAGAGCACTCGGCTGTTGGGTGTGGTTCAGGAAGCTCACTGGCAGAGAGTGTCTGTGGCCCCAGTCAAGGTTTTTACCCAGTGACAGATAACTGGAAATGACAATGTTGATAGGAAATAAAGTGTGGTGCCACCATGGAGTCATCCCTGGCCACTCCCGACACAAGCGAGGCTTGACTATACTACTGTGGAGTATACACGTGCAATAATCAGTGAACTCAATCCCTGGTGGACAGAAGAGCCGCCCAAGCTGCGTGTAATCTCTGGGTGCTATCACAGTGCGTTTCACGAGTGCTGTTTCCAGGCAGTTAGGTTGGGCTGAACTTGTGAGTATCAAACAGTGCTCTTCTTTCTGTGTAGACCTTCAGCAAAAGCAGGTACTTGGAAACCACAGGCTCACCTTCTCTATCTATTCAGTAATTATTAACAAAGAGACCTCCATAAGGGAGCACTTGGCTGTTACTGATAATGTACCAATTGCTAAAGAACCCGTCTCCAAGCCATCCGGTGATGTGTGCAGCATCACTGTAGAACTGTCCCGTGCCGCTCTTCACTTCCCTTTGGGTGGAGCCTTTTCGACTCCCCATGGCAGGCTAATCTTTCTCTCCAGGTAGTAACTTGGCCCCGAGGTTGGGCAAGTACTCCCCAGACTGAGAGAAGCGGCTGCGTCACATCTGCTGTGTTCCGTCATTGGTGATCAGCCAGTCAGCATTCGCTCCCTGTGTCTAACTCTCTCTCTCGCACCTCTCCCAGATGCTATCTATCGGGTTTTCTCACTGTTGCCAGTGGATGTCATCCAAACAGTGGGCTCATATCTTATGGTTTTTGTGCAATCATTGTCGTATTGTAGTCCTAAGACTCATTATAGTGTATTTTTGATATTTTTGAAATGTGTTAAATTTTTTAATTCAATAATATGAGCCAGAGCATGTTGCAGCAAATCTATTGTTTGTAAAAAATAATAATAAACAATAAATAAAATAAAATGGGATATCTTTTCCA

(**1**) Phlpp2 3’UTR fragments cloned for reporter assay

**Fragment Start End**

Phlpp2 971 1687

(**2**) Predicted miRNA binding sites

**miRNA site Position Sequence**

miR-19 1445-1451 TTGCACA
